# Major Bleeding Risk in Patients With Non-valvular Atrial Fibrillation Concurrently Taking Direct Oral Anticoagulants and Antidepressants

**DOI:** 10.3389/fnagi.2022.791285

**Published:** 2022-02-04

**Authors:** Kuo-Hsuan Chang, Chiung-Mei Chen, Chun-Li Wang, Hui-Tzu Tu, Yu-Tung Huang, Hsiu-Chuan Wu, Chien-Hung Chang, Shang-Hung Chang

**Affiliations:** ^1^Department of Neurology, Chang Gung Memorial Hospital, Linkou Medical Center, Taoyuan, Taiwan; ^2^College of Medicine, Chang Gung University, Taoyuan, Taiwan; ^3^Division of Cardiology, Department of Internal Medicine, Chang Gung Memorial Hospital, Linkou Medical Center, Taoyuan, Taiwan; ^4^Center for Big Data Analytics and Statistics, Chang Gung Memorial Hospital, Linkou Medical Center, Taoyuan, Taiwan; ^5^Graduate Institute of Nursing, Chang Gung University of Science and Technology, Taoyuan, Taiwan

**Keywords:** direct oral anticoagulants, antidepressants, atrial fibrillation, intracerebral hemorrhage, gastrointestinal bleeding

## Abstract

Direct oral anticoagulants (DOACs) are commonly prescribed with antidepressants that may increase bleeding risk. Here we assessed the association between DOACs with and without concurrent antidepressants and major bleeding risk in patients with atrial fibrillation (AF) by a retrospective cohort study included patients with AF who received prescriptions of DOACs in Taiwan’s National Health Insurance database between 2012 and 2017. Adjusted rate ratio (ARR) of major bleeding was calculated by comparing incidence rate adjusted with Poisson regression and inverse probability of treatment weighting using the propensity score between patient-times with and without antidepressants. Among 98863 patients with AF, concurrent use of bupropion with DOACs increased the risks of all major bleeding (ARR: 1.49, 95% CI: 1.02–2.16) and gastrointestinal hemorrhage (ARR: 1.57, 95% CI: 1.04–2.33). An increased risk of intracerebral hemorrhage (ICH) was associated with the combinations of DOACs with selective serotonin reuptake inhibitors (SSRIs, ARR: 1.38, 95% CI: 1.08–1.76), particularly in paroxetine (ARR: 2.11, 95% CI: 1.17–3.81), and tetracyclic antidepressants (TeCAs, ARR: 1.34, 95% CI: 1.01–1.78). In subgroup analyses stratified by individual NOACs, SSRIs increased the risk of ICH in the dabigatran-treated patients (ARR: 1.55, 95% CI: 1.04–2.33). The combinations of apixaban and serotonin-norepinephrine reuptake inhibitors (SNRIs) were associated with a higher risk of all major bleeding (ARR: 1.63, 95% CI: 1.04–2.55). These results clearly indicate the drug–drug interactions between DOACs and antidepressants, which should be carefully considered when prescribing DOACs in adult patients. Careful monitoring for bleeding should be performed while concurrently prescribing DOACs with bupropion, SSRI, SNRI, and TeCA. Concomitant use of DOACs and TCAs may be a relatively safe strategy for patients with AF.

## Introduction

Atrial fibrillation (AF) is a prominent cause of ischemic stroke, and oral anticoagulation is frequently indicated for stroke prevention in patients with non-valvular AF (Rasmussen et al., 2012). Direct antagonist oral anticoagulants (DOACs) and warfarin are indicated for the prevention of stroke and systemic embolism in AF and for the treatment of venous thromboembolism (Zirlik and Bode, 2017). Over the past few years, four DOACs, apixaban, rivaroxaban, dabigatran, and edoxaban, have been shown to offer several advantages over warfarin (Yeh et al., 2015). These DOACs have a rapid onset of therapeutic action, shorter half-life, and predictable pharmacodynamic effects; and do not require routine laboratory monitoring (Mekaj et al., 2015). However, DOACs still pose clinically relevant bleeding risks, especially in patients with multiple comorbidities, those with polypharmacy, or those using high-risk medications (Chang et al., 2017). Significant drug–drug interactions are more likely to occur when DOACs are co-administered with medications that change the activity of the multidrug efflux transporter permeability glycoprotein (P-gp) or cytochrome P450 3A4 (CYP3A4) system (Steffel et al., 2018). Medications that inhibit CYP3A4 or P-gp activities, such as amiodarone and fluconazole, may increase DOAC levels and the bleeding risk (Chang et al., 2017; Wang et al., 2020). Avoidance of concurrent prescribing of these medications with DOACs would be necessary for patients with high risk of bleeding.

Depression, a common mental illness in the patients with stroke, is associated with an increased disability, increased cognitive impairment, increased mortality, and worse rehabilitation outcome (Gillen et al., 2001; House et al., 2001; Pohjasvaara et al., 2001; Halperin and Reber, 2007). Antidepressants, such as selective serotonin reuptake inhibitors (SSRIs), selective serotonin–norepinephrine reuptake inhibitors (SNRIs), tricyclic antidepressants (TCAs), and tetracyclic antidepressants (TeCAs), are widely used in the treatment of depression, especially among patients with stroke (El Husseini et al., 2012). However, these medications may affect platelet function, increasing the risk of bleeding (Verdel et al., 2011). For example, SSRIs reduce the ability of platelets to aggregate and increase the risk of bleeding (Skop and Brown, 1996). Clinically, exposure to SSRIs is also associated with an increased risk of gastrointestinal and intracerebral hemorrhage (ICH) (de Abajo et al., 1999; Dalton et al., 2003; Meijer et al., 2004; Hackam and Mrkobrada, 2012). The odds ratios of gastrointestinal hemorrhage in patients taking SSRIs varied from 1.38 to 3.60 (de Abajo et al., 1999; Dalton et al., 2003; Meijer et al., 2004). A meta-analysis also showed the increased rate ratio (1.42) of ICH in patients taking SSRIs (Hackam and Mrkobrada, 2012). Of note, many antidepressants interact with P-gp and CYP3A4 as substrates or inhibitors (DeVane et al., 2004; O’Brien et al., 2012). An *in vitro* study has demonstrated that paroxetine is a substrate of P-gp (Weiss et al., 2003). Co-administration of a P-gp inhibitor, itraconazole, increases the plasma concentration of paroxetine (Yasui-Furukori et al., 2007). Norfluoxetine, the metabolite of fluoxetine, has an inhibitory effect on CYP3A4 (Weiss et al., 2003). However, little is known regarding the clinical relevance of these drug–drug interactions. Previous studies to explore the effects of antidepressants on bleeding risk in patients taking DOACs also demonstrate conflict results (Sheikh Rezaei et al., 2019; Komen et al., 2020; Marchena et al., 2020). To address this important question, we conducted a nationwide retrospective cohort study to compare the major bleeding risk of patients with AF concurrently treated with DOACs and antidepressants and those who took DOACs alone.

## Materials and Methods

### Research Ethics

This retrospective cohort study was approved by the Institutional Review Board of Chang Gung Memorial Hospital, Linkou, Taiwan and complied fully with existing national ethical and regulatory guidelines (ethical license no: 201901357B0). The need to provide written informed consent was waived by the ethics committee because all data were anonymized by the Taiwan National Health Insurance (NHI) Administration.

### Data Source

The data of patients were retrieved from the NHI Research Database and included outpatient, inpatient, and prescription records accessed through the Applied Health and Welfare Data Science Center, Ministry of Health and Welfare in Taiwan. Under a single payer system operated by the Taiwanese government, NHI covered 99% of Taiwan’s population. After the billing process, the National Health Research Institutes (NHRI) compiles data from these insurance claims for research purposes. All identifying information was encrypted. Diagnoses/procedures were identified using International Classification of Diseases, 9th Revision, Clinical Modification (ICD-9-CM) codes from 1997 to 2015 and International Classification of Diseases, 10th Revision, Clinical Modification (ICD-10-CM) codes since 2016.

### Study Population

All patients (outpatients and/or inpatients) with 2 or more consecutive records of AF diagnosis (ICD-9-CM code 427.31 or ICD-10-CM code I48) and DOAC (dabigatran, rivaroxaban, apixaban, or edoxaban) prescriptions for more than 28 days from June 1, 2012, to December 31, 2017, were included. The index date was defined as the first DOAC prescription. Patients were excluded if they took DOACs for indications other than AF, including pulmonary embolism, deep vein thrombosis, mitral stenosis, joint replacement, or valvular surgery within 6 months before the index date, had end-stage renal disease, or were aged less than 30 years or greater than 105 years. Patients were followed up until death, withdrawal from the NHI, or the end of the study period (December 31, 2017).

### Follow-Up of Patients

We divided each calendar year into 4 quarters for each patient. The analytic unit was one person-quarter. Person-quarters were used because medications for chronic illnesses were refilled with a maximum length of 3 months under the NHI reimbursement policy. Medications and covariates were assessed for each person-quarter, which simplified the assessment of the complex prescription pattern of DOACs and concurrent drugs (Supplementary Figure 1). We identified person-quarters exposed to DOACs with or without concurrent medications. Person-quarters fully prescribed with DOACs and/or antidepressants within the indicated quarter were assigned as those exposed to specific medications. The major bleeding risk of person-quarters exposed to DOACs and concurrent antidepressants was compared with person-quarters exposed to DOACs alone. Only antidepressants used by more than 200 patients were enrolled. These antidepressants were further classified as SNRI (duloxetine or venlafaxine), SSRI (citalopram, escitalopram, fluoxetine, paroxetine, or sertraline), TCA (amitriptyline, imipramine, or melitracen), TeCA (mirtazapine or trazodone), and others (agomelatine and bupropion). Person-quarters with concomitant prescriptions of DOACs and warfarin, or two DOACs were excluded. Each antidepressant was calculated separately in person-quarters with multiple antidepressants (0.61% person-quarters).

### Major Outcomes

The primary outcome was major bleeding, which was identified by ICD codes at the emergency department visit with a primary diagnosis of ICH, gastrointestinal or other bleedings including intraspinal, intraocular, retroperitoneal, intra-articular, pericardial, or intramuscular hemorrhage (Table 1 and Supplementary Table 1). Patients with traumatic hemorrhage were excluded.

**TABLE 1:** Demographic analysis of patients taking direct oral anticoagulants (DOACs).

	DOAC users (*n* = 98 863)
Age (years) (range)	74.89 ± 10.32 (30–98)
Male (%)	55 610 (56.25%)
CHAD_2_DS_2_-VASc score (range)	4.69 ± 1.83 (2–9)
Anemia (%)	15 520 (15.7%)
Acute pancreatitis (%)	1639 (1.66%)
Acute appendicitis (%)	1601 (1.62%)
Cancer (%)	14 064 (14.23%)
Metastatic solid tumor (%)	1334 (1.35%)
**Cardiovascular diseases**	
Hypertension (%)	86 688 (87.68%)
Myocardial infarction (%)	6507 (6.58%)
Congestive heart failure (%)	51 300 (51.89%)
Peripheral vascular disease (%)	13 896 (14.06%)
Peripheral arterial occlusive disease (%)	2677 (2.71%)
Percutaneous coronary intervention (%)	8660 (8.76%)
Coronary artery bypass surgery (%)	1121 (1.13%)
Chronic kidney disease (%)	26 568 (26.87%)
**Gastrointestinal and hepatic diseases**	
Peptic ulcer disease (%)	56 455 (57.10%)
Mild liver disease (%)	36 580 (37.00%)
Moderate or severe liver disease (%)	334 (0.34%)
Human immunodeficiency virus infection (%)	25 (0.03%)
Intestinal obstruction without mention of hernia (%)	5147 (5.21%)
**Metabolic disease**	
Diabetes Mellitus (%)	41 790 (42.27%)
Diabetes with complications (%)	14 745 (14.91%)
**Neurological diseases**	
Cerebral vascular disease (%)	50 422 (51.00%)
Ischemic stroke (%)	37 610 (38.04%)
Transient ischemic attack (%)	12 915 (13.06%)
Hemiplegia and paraplegia (%)	5096 (5.15%)
Dementia (%)	11 427 (11.56%)
Epilepsy (%)	2793 (2.83%)

### Covariates

Demographics, comorbidities, medications, and medical expense for each person-quarter relevant to the first date of enrollment of patients were identified as covariates. Demographics included age, sex, income level, residency, occupation, and number of outpatient visits. The CHA2DS2-VASc score (Lip et al., 2010) and diseases probably related to hemorrhage were also added as covariates (Table 1 and Supplementary Tables 2, 3). Medications probably related to hemorrhage or interacting with DOACs and prescribed longer than 28 days in each person-quarter were also recognized as covariates (Table 2 and Supplementary Table 4).

**TABLE 2:** Medications during follow-up.

	DOAC users (*n* = 98 863)
Antibiotics and antifungal drugs	2800 (2.83%)
Anticoagulants	98 863 (100%)
Apixaban	20 825 (21.06%)
Dabigatran	42 779 (43.27%)
Edoxaban	10 517 (10.64%)
Rivaroxaban	55 587 (56.23%)
Antidepressants	19 638 (19.86%)
SNRI	2087 (2.11%)
Duloxetine	1536 (1.55%)
Venlafaxine	626 (0.63%)
SSRI	7234 (7.32%)
Citalopram	3695 (3.74%)
Escitalopram	3442 (3.48%)
Fluoxetine	934 (0.94%)
Paroxetine	875 (0.89%)
Sertraline	2751 (2.78%)
TCA	9591 (9.70%)
Amitriptyline	3183 (3.22%)
Imipramine	6177 (6.25%)
Melitracen	3183 (3.22%)
TeCA	6149 (6.22%)
Mirtazapine	1656 (1.68%)
Trazodone	4922 (4.98%)
Others	1179 (1.19%)
Agomelatine	653 (0.66%)
Bupropion	534 (0.54%)
Antiepileptics	4928 (4.98%)
Antihypertensives	59 324 (60.10%)
Antiplatelets	24 186 (24.46%)
Cardiovascular drugs	36 505 (36.92%)
Cyclosporine (%)	49 (0.05%)
Glucocorticoid (%)	7987 (8.08%)
Insulin (%)	6236 (6.31%)
Lipid lowering drugs	19 172 (19.39%)
Non-steroid anti-inflammatory drugs (%)	19 180 (19.40%)

*DOAC, direct oral anticoagulant; SNRI, selective serotonin–norepinephrine reuptake inhibitor; SSRI, selective serotonin reuptake inhibitors; TCA, tricyclic antidepressant; TeCA, tetracyclic antidepressant.*

### Propensity Score Weighting

Confounding by indication from non-random treatment allocation for concurrent medications was a crucial feature when comparing bleeding risk between patients treated with DOACs with and without concurrent antidepressants. This bias was accounted for by the propensity score, which estimated the probability that a patient was prescribed the concurrent antidepressant during a person-quarter. The specific propensity score for each antidepressant was calculated by applying logistic regression to the identified covariates (Tables 1, 2 and Supplementary Tables 2–4) relevant to the first date of the person-quarter. The distribution of propensity scores in each group stratified by DOACs and antidepressants did not present a significant number of outliers indicative of extreme weight (data not shown). Standardized differences were estimated to assess the balance of individual covariates before and after inverse probability of treatment weighting using propensity score (Supplementary Tables 5–9). A negligible difference was defined as an absolute standardized mean difference of <0.1.

### Statistical Analysis

To account for the intraindividual correlation across person-quarters, we used Poisson regression with a generalized estimating equation model to calculate the adjusted incidence rate that considered the inverse probability of treatment weighting by propensity scores if the number of bleeding events was greater than 3. All regression analyses were performed separately for each combination/medication with more than three bleeding events; person-quarters using DOAC alone was used as the reference. To preclude the potentially unmeasured confounders (Lipsitch et al., 2010), we used acute pancreatitis and acute appendicitis as negative control outcomes. To evaluate the potential reverse causality, we performed a sensitivity analysis to remove the bleeding events that occurred within the first person-quarter following prescriptions of DOACs or antidepressants. Patients with missing data (<0.1% of DOAC users) were excluded. The analysis was conducted using SAS (SAS Institute), version 9.4.

## Results

**FIGURE 1:**
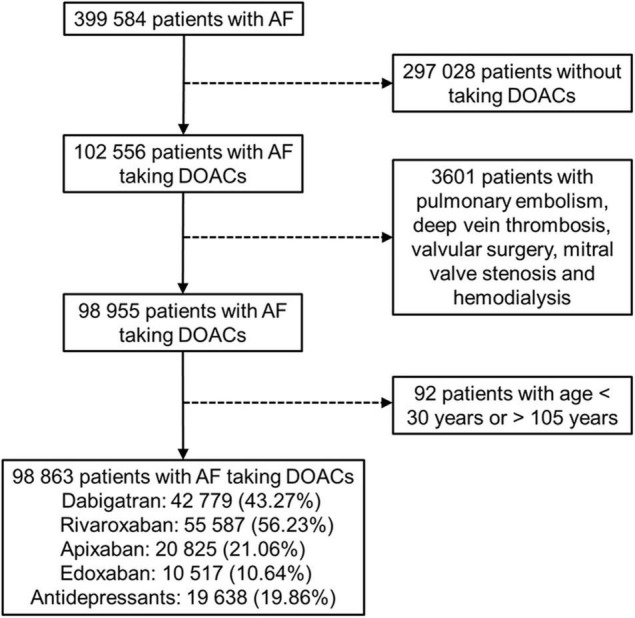
Enrollment of patients with non-valvular atrial fibrillation (AF) using direct oral anticoagulants (DOACs).

We identified a total of 98 863 patients with AF who received DOAC therapy between June 1, 2012, and December 31, 2017 (Figure 1). The characteristics of the patients at the first date of DOAC prescription are listed in Tables 1, 2 and Supplementary Tables 3, 4. The mean age was 74.89 ± 10.32 years, and 56.25% of the studied population comprised men. The baseline average CHA2DS2-VASc score was 4.69 ± 1.83. Hypertension was observed in 87.68% of the included patients, and congestive heart failure and cerebrovascular disease were noted in 50.89% and 51.00% of the included patients, respectively. Diabetes mellitus was diagnosed in 42.27% of the included patients. A total of 19 638 (19.86%) DOAC-treated patients with AF concurrently used antidepressants (Figure 1). The most common antidepressants prescribed with concurrent DOACs were imipramine (6.25%), followed by trazodone (4.98%), citalopram (3.74%), and escitalopram (3.48%).

During follow-up, 6297 (6.37%) patients had one major bleeding events. Multiple major bleeding events occurred in 782 (0.80%) patients. Among 705 521 person-quarters with DOAC prescriptions, 8037 (1.14%) major bleeding events were identified. 71 374 (10.12%) person-quarters were prescribed with antidepressants. Multiple antidepressants were prescribed in 4333 (0.61%) person-quarters. Table 3 and Supplementary Table 10 summarized the incidence rate and adjusted incidence rate for major bleeding events among the 14 combinations of a DOAC and an antidepressant. Although there was no subclass of antidepressants increased bleeding risk among the DOAC-treated patients with AF, the individual drug analysis showed that the combination of DOACs with bupropion was significantly associated with a higher risk of major bleeding compared with DOACs alone (adjusted rate ratio [ARR]: 1.49, 95% confidence interval [CI]: 1.02–2.16, *P* = 0.037). The combinations of DOACs with any of the other antidepressants were not associated with a higher risk of major bleeding. Subgroup analysis by bleeding site revealed an increased risk of ICH associated with the combinations of DOACs with SSRIs (ARR: 1.38, 95% CI: 1.08–1.76, *P* = 0.010) and TeCA (ARR: 1.34, 95% CI: 1.01–1.78, *P* = 0.041) compared with DOACs alone in patients with AF (Table 4 and Supplementary Table 11). Individual drug analysis determined that the combination of paroxetine (ARR: 2.11, 95% CI: 1.17–3.81, *P* = 0.013) with DOACs in the patients with AF were associated with a higher risk of ICH compared with DOACs alone. Bupropion increased the risk of gastrointestinal hemorrhage in the DOAC-treated patients with AF (ARR: 1.57, 95% CI: 1.04–2.35, *P* = 0.031, Table 5 and Supplementary Table 12). None of the antidepressants were associated with a high risk of intraspinal, intraocular, retroperitoneal, intra-articular, pericardial, or intramuscular hemorrhage (Supplementary Table 13). Separate analyses showed that SSRIs increased the risk of ICH in the dabigatran-treated patients with AF (ARR: 1.55, 95% CI: 1.04–2.33, *P* = 0.033, Table 6 and Supplementary Table 14). The combinations of apixaban and SNRIs were associated with a higher risk of major bleeding (ARR: 1.63, 95% CI: 1.04–2.55, *P* = 0.032, Table 6 and Supplementary Table 15). None of antidepressants increased the risk of bleeding in AF patients treated with rivaroxaban (Supplementary Table 16). Because edoxaban was introduced in Taiwan after 2016, we did not include it in the separate analysis.

**TABLE 3:** Major bleeding risk among patients with atrial fibrillation (AF) taking DOACs with or without concurrent antidepressants.

Concurrent medication	Person-Quarters with DOAC use	No. of Bleeding Events	Crude Major Bleeding Incidence Rate (95% CI) per 1000 Person-Years		*Adjusted Incidence Rate (95% CI) per 1000 Person-Years		*Adjusted Rate Ratio (95% CI)	
**SNRI**								
With	6642	97	58.84	(47.80–72.43)	59.25	(48.22–72.79)	1.15	(0.93–1.42)
†without	698 879	7940	45.93	(44.83–47.06)	51.47	(49.06–54.01)	1	
**SSRI**								
With	25 344	329	51.86	(46.29–58.09)	52.47	(46.90–58.71)	1.02	(0.91–1.15)
†without	680 177	7708	45.83	(44.72–46.97)	51.45	(49.92–53.01)	1	
**TCA**								
With	25 083	319	50.99	(45.44–57.21)	51.4	(45.84–57.63)	1.02	(0.91–1.15)
†without	680 438	7718	45.87	(44.75–47.01)	50.38	(48.92–51.90)	1	
**TeCA**								
With	19 179	267	55.68	(49.05–63.21)	56.42	(49.76–63.98)	1.07	(0.95–1.22)
†without	686 342	7770	45.78	(44.67–46.91)	52.51	(51.03–54.04)	1	
**Others**								
With	3511	41	47.02	(34.35–64.36)	47.44	(34.79–64.68)	0.97	(0.71–1.33)
†without	702 010	7996	46.04	(44.94–47.17)	48.86	(47.40–50.36)	1	
**Bupropion**								
With	1457	26	71.61	(49.25–104.11)	71.8	(49.55–104.04)	#1.49	(1.02–2.16)
†without	704 064	8011	46	(44.90–47.12)	48.33	(46.75–49.97)	1	

*Adjusted by inverse probability of treatment weighting using the propensity score (gender, age, medical utilization, hypertension, myocardial infarction, congestive heart failure, percutaneous coronary intervention, coronary bypass surgery, peripheral vascular disease, cerebrovascular disease, ischemic stroke, transient ischemic attack, hemiplegia or paraplegia, dementia, epilepsy, diabetes mellitus, chronic kidney disease, chronic pulmonary disease, peptic ulcer disease, liver disease, malignancy, anemia, rheumatic disease, human immunodeficiency virus infection, antibiotics and antifungal drugs, antiepileptics, antihypertensives, antiplatelets, bisphosphate, cardiovascular drugs, cyclosporine, glucocorticoid, insulin, lipid lower drugs, non-steroid anti-inflammatory drugs, residence, income level, and occupation; see Tables 1, 2 and Supplementary Tables 2–4).

*†“Without” indicates DOACs alone.*

*#P < 0.05, compared with DOACs alone.*

*DOAC, direct oral anticoagulant; SNRI, selective serotonin–norepinephrine reuptake inhibitor; SSRI, selective serotonin reuptake inhibitors; TCA, tricyclic antidepressant; TeCA, tetracyclic antidepressant.*

**TABLE 4:** Risk of intracerebral hemorrhage among patients with AF taking DOACs with or without concurrent antidepressants.

Concurrent medication	Person-Quarters with DOAC use	No. of Bleeding Events	Crude Major Bleeding Incidence Rate (95% CI) per 1000 Person-Years		*Adjusted Incidence Rate (95% CI) per 1000 Person-Years		*Adjusted Rate Ratio (95% CI)	
**SNRI**								
With	6642	14	8.68	(5.16–14.59)	8.55	(5.07–14.44)	1.1	(0.65–1.87)
†without	698 879	1250	7.23	(6.83–7.66)	7.78	(7.21–8.39)	1	
**SSRI**								
With	25 344	75	11.87	(9.39–15.00)	12.01	(9.52–15.15)	#1.38	(1.08–1.76)
†without	680 177	1189	7.07	(6.67–7.51)	8.73	(8.09–9.41)	1	
**TCA**								
With	25 083	54	8.69	(6.66–11.34)	8.66	(6.64–11.30)	1.18	(0.90–1.55)
without	680 438	1210	7.19	(6.78–7.63)	7.33	(6.88–7.82)	1	
**TeCA**								
With	19 179	52	10.88	(8.24–14.36)	10.94	(8.30–14.42)	#1.34	(1.01–1.78)
†without	686 342	1212	7.15	(6.74–7.58)	8.15	(7.61–8.72)	1	
**Others**								
With	3511	5	5.71	(2.34–13.94)	5.79	(2.41–13.91)	0.71	(0.29–1.71)
†without	702 010	1259	7.26	(6.85–7.69)	8.18	(7.56–8.85)	1	
**Paroxetine**								
With	2601	11	16.95	(9.45–30.39)	16.9	(9.41–30.36)	#2.11	(1.17–3.81)
†without	702 920	1253	7.21	(6.81–7.64)	8	(7.43–8.61)	1	

*Adjusted by inverse probability of treatment weighting using the propensity score (gender, age, medical utilization, hypertension, myocardial infarction, congestive heart failure, percutaneous coronary intervention, coronary bypass surgery, peripheral vascular disease, cerebrovascular disease, ischemic stroke, transient ischemic attack, hemiplegia or paraplegia, dementia, epilepsy, diabetes mellitus, chronic kidney disease, chronic pulmonary disease, peptic ulcer disease, liver disease, malignancy, anemia, rheumatic disease, human immunodeficiency virus infection, antibiotics and antifungal drugs, antiepileptics, antihypertensives, antiplatelets, bisphosphate, cardiovascular drugs, cyclosporine, glucocorticoid, insulin, lipid lower drugs, non-steroid anti-inflammatory drugs, residence, income level, and occupation; see Tables 1, 2 and Supplementary Tables 2–4).

*†“Without” indicates DOAC alone.*

*#P < 0.05, compared with DOAC alone.*

*DOAC, direct oral anticoagulant; SNRI, selective serotonin–norepinephrine reuptake inhibitor; SSRI, selective serotonin reuptake inhibitors; TCA, tricyclic antidepressant; TeCA, tetracyclic antidepressant.*

**TABLE 5:** Risk of gastrointestinal hemorrhage among patients with AF taking DOACs with or without concurrent antidepressants.

Concurrent medication	Person- Quarters with DOAC use	No. of Bleeding Events	Crude Major Bleeding Incidence Rate (95% CI) per 1000 Person-Years		*Adjusted Incidence Rate (95% CI) per 1000 Person-Years		*Adjusted Rate Ratio (95% CI)	
**SNRI**								
With	6642	79	47.68	(37.76–60.21)	48.18	(38.27–60.67)	1.15	(0.90–1.45)
†without	698 879	6411	37.03	(36.04–38.06)	42.05	(39.73–44.51)	1	
**SSRI**								
With	25 344	248	39	(34.20–44.48)	39.47	(34.66–44.95)	0.96	(0.84–1.10)
†without	680 177	6242	37.06	(36.06–38.10)	41.06	(39.70–42.46)	1	
**TCA**								
With	25 083	260	41.42	(36.38–47.15)	41.85	(36.80–47.59)	1.01	(0.89–1.15)
without	680 438	6230	36.98	(35.97–38.01)	41.4	(40.02–42.82)	1	
**TeCA**								
With	19 179	206	42.93	(37.22–49.51)	43.46	(37.74–50.06)	1.02	(0.88–1.17)
without	686 342	6284	36.97	(35.97–38.00)	42.73	(41.38–44.13)	1	
**Others**								
With	3511	36	41.34	(29.55–57.84)	41.59	(29.84–57.97)	1.07	(0.76–1.49)
without	702 010	6454	37.11	(36.12–38.14)	39	(37.72–40.33)	1	
**Bupropion**								
With	1457	22	60.23	(39.94–90.84)	60.65	(40.47–90.89)	#1.57	(1.04–2.35)
without	704 064	6468	37.09	(36.09–38.11)	38.74	(37.29–40.25)	1	

*Adjusted by inverse probability of treatment weighting using the propensity score (gender, age, medical utilization, hypertension, myocardial infarction, congestive heart failure, percutaneous coronary intervention, coronary bypass surgery, peripheral vascular disease, cerebrovascular disease, ischemic stroke, transient ischemic attack, hemiplegia or paraplegia, dementia, epilepsy, diabetes mellitus, chronic kidney disease, chronic pulmonary disease, peptic ulcer disease, liver disease, malignancy, anemia, rheumatic disease, human immunodeficiency virus infection, antibiotics and antifungal drugs, antiepileptics, antihypertensives, antiplatelets, bisphosphate, cardiovascular drugs, cyclosporine, glucocorticoid, insulin, lipid lower drugs, non-steroid anti-inflammatory drugs, residence, income level, and occupation; see Tables 1, 2 and Supplementary Tables 2–4).

*†“Without” indicates DOACs alone.*

*#P < 0.05, compared with DOACs alone.*

*DOAC, direct oral anticoagulant; SNRI, selective serotonin–norepinephrine reuptake inhibitor; SSRI, selective serotonin reuptake inhibitors; TCA, tricyclic antidepressant; TeCA, tetracyclic antidepressant.*

**TABLE 6:** Risk of major bleeding among patients with AF taking dabigatran/apixaban with or without concurrent antidepressants.

Concurrent medication	Person- Quarters with DOAC use	No. of Bleeding Events	Crude Major Bleeding Incidence Rate (95% CI) per 1000 Person-Years		*Adjusted Incidence Rate (95% CI) per 1000 Person-Years		*Adjusted Rate Ratio (95% CI)	
Major bleeding (Dabigatran)								
**SNRI**								
with	2665	39	59.57	(43.42–81.72)	59.13	(43.14–81.04)	1.32	(0.96–1.83)
†without	267 899	2678	40.49	(38.87–42.17)	44.64	(41.81–47.66)	1	
**SSRI**								
with	9680	119	49.27	(40.70–59.65)	49.77	(41.17–60.17)	1.09	(0.90–1.32)
†without	260 884	2598	40.35	(38.72–42.05)	45.77	(43.58–48.08)	1	
**TCA**								
with	10242	116	45.58	(37.80–54.96)	45.65	(37.87–55.03)	1.05	(0.86–1.27)
†without	260 322	2601	40.48	(38.84–42.18)	43.61	(41.64–45.68)	1	
**TeCA**								
with	7252	98	54.2	(43.76–67.12)	54.62	(44.16–67.57)	1.19	(0.95–1.47)
†without	263 312	2619	40.3	(38.68–41.98)	46.09	(43.90–48.39)	1	
**Others**								
with	1293	12	36.75	(20.00- 67.54)	37.44	(20.56- 68.19)	0.9	(0.49 - 1.65)
†without	269 271	2705	40.69	(39.08- 42.38)	41.48	(39.31- 43.78)	1	
**Intracerebral hemorrhage (Dabigatran)**								
**SSRI**								
with	9680	26	10.78	(7.31–15.90)	10.9	(7.41–16.03)	#1.55	(1.04–2.33)
†without	260 884	364	5.64	(5.07–6.26)	7.01	(6.15–8.00)	1	
**Major bleeding (Apixaban)**								
**SNRI**								
with	813	19	95.03	(61.46–146.94)	94.32	(60.87–146.17)	#1.63	(1.04–2.55)
†without	89 336	1079	49.05	(46.00–52.30)	57.76	(50.47–66.11)	1	
**SSRI**								
with	3385	44	52.39	(38.97–70.42)	52.48	(39.14–70.36)	0.95	(0.70–1.28)
†without	86 764	1054	49.34	(46.24–52.65)	55.52	(51.12–60.29)	1	
**TCA**								
with	3127	43	55.22	(40.63–75.05)	55.57	(41.01–75.30)	1.1	(0.80–1.50)
†without	87 022	1055	49.25	(46.15–52.55)	50.74	(47.19–54.55)	1	
**TeCA**								
with	2477	39	63.62	(45.97–88.03)	63.74	(46.04–88.23)	1.12	(0.80–1.57)
†without	87 672	1059	49.06	(45.98–52.33)	56.75	(52.43–61.43)	1	
**Others**								
with	511	6	46.19	(20.27–105.24)	47.32	(21.32–105.02)	0.86	(0.39–1.92)
†without	89 638	1092	49.47	(46.41–52.73)	54.93	(50.03–60.31)	1	

*Adjusted by inverse probability of treatment weighting using the propensity score (gender, age, medical utilization, hypertension, myocardial infarction, congestive heart failure, percutaneous coronary intervention, coronary bypass surgery, peripheral vascular disease, cerebrovascular disease, ischemic stroke, transient ischemic attack, hemiplegia or paraplegia, dementia, epilepsy, diabetes mellitus, chronic kidney disease, chronic pulmonary disease, peptic ulcer disease, liver disease, malignancy, anemia, rheumatic disease, human immunodeficiency virus infection, antibiotics and antifungal drugs, antiepileptics, antihypertensives, antiplatelets, bisphosphate, cardiovascular drugs, cyclosporine, glucocorticoid, insulin, lipid lower drugs, non-steroid anti-inflammatory drugs, residence, income level, and occupation; see Tables 1, 2 and Supplementary Tables 2–4).

*†“Without” indicates dabigatran or apixaban alone.*

*#P < 0.05, compared with dabigatran or apixaban alone.*

*DOAC, direct oral anticoagulant; SNRI, selective serotonin–norepinephrine reuptake inhibitor; SSRI, selective serotonin reuptake inhibitors; TCA, tricyclic antidepressant; TeCA, tetracyclic antidepressants.*

None of the combinations were associated with a high risk of unrelated events as negative control outcomes, such as acute pancreatitis or acute appendicitis (Supplementary Table 17). The results of evaluating the potential reverse causality by removing the bleeding events that occurred within the first person-quarter was similar to the main findings (Supplementary Table 18).

## Discussion

This nationwide population-based cohort study determined that bupropion was associated with a significantly increased risk of major bleeding, particularly gastrointestinal bleeding. SSRIs, particularly paroxetine, and TeCAs were associated with a significantly increased risk of ICH. Bleeding risk varied among DOACs. In AF patients taking dabigatran, SSRIs also increased the risk of ICH. SNRIs were associated with an increased risk of major bleeding in apixaban-treated AF patients. TCAs were not associated with an increased risk of any major bleeding. Concomitant use of DOACs and TCAs may be a relatively safe strategy for patients with AF.

Our results uncovered an association between bupropion and an increased risk of major bleeding and gastrointestinal hemorrhage in the patients with AF taking DOACs. Bupropion is a potent inhibitor of CYP2D6 (Kotlyar et al., 2005), whereas its effect on CYP3A4 remains uncertain. An *in vitro* study demonstrated that bupropion and its metabolites exhibited only little affinity for P-gp (Wang et al., 2008). Therefore, the mechanism of these associations remains elusive. Of note, bupropion have been linked to cases of hepatic injury, which could increase the risk of bleeding (Voican et al., 2014). An unknown drug–drug interaction by polypharmacy in patients with AF may also contribute to the increased risk of bleeding. Careful monitoring for bleeding would be necessary in patients with AF concomitantly taking DOACs and bupropion.

The association between ICH and SSRIs has been demonstrated in several observational studies (Hackam and Mrkobrada, 2012; Renoux et al., 2017). Serotonin stored in platelets represents more than 99% of the total serotonin concentration in the human body (Li et al., 1997). After vascular injury and platelet activation, serotonin is released into the bloodstream and binds to specific receptors to promote vasoconstriction and platelet aggregation and thereby hemostasis (Li et al., 1997). Reuptake of serotonin into platelets involves a serotonin transporter that is blocked by SSRIs (Halperin and Reber, 2007). This inhibition would in turn reduce the potential for platelet aggregation and therefore platelet thrombus formation, with a subsequent increased risk of bleeding. SSRIs with a larger degree of serotonin reuptake inhibition have been more frequently associated with abnormal bleeding and modification of hemostasis markers (Halperin and Reber, 2007). The risk of ICH in patients exposed concurrently to anticoagulants and SSRIs has not been extensively explored. In a case–control study, SSRIs and warfarin did not confer an increased risk of ICH compared with warfarin alone (Kharofa et al., 2007). Our study demonstrated a higher risk of ICH in the patients with AF concurrently taking DOACs and SSRIs compared with those taking DOACs alone. The individual drug analysis identified associations between paroxetine and an increased risk of ICH in this patient group. Notably, paroxetine demonstrates a high degree of serotonin reuptake inhibition (Tatsumi et al., 1997; Renoux et al., 2017) and could be a substrate of CYP3A4 and P-gp (Cozza et al., 2003; O’Brien et al., 2012). The competitive binding to CYP3A4 by paroxetine may enhance the effect of DOACs. However, P-gp-mediated drug efflux at the blood-brain barrier may limit drug delivery to the brain (Loscher and Potschka, 2005). Competitive binding to P-gp by DOACs may prevent the drug efflux by P-gp at the blood-brain barrier and increase the brain concentration of these SSRIs, leading to an increased risk of ICH.

Separated drug analysis further showed that SSRIs increased risk of ICH in AF patients taking dabigatran. Dabigatran is one of the most frequently involved drugs for the occurrence of drug adverse-effects (Shehab et al., 2016). Dabigatran is contraindicated with potent P-gp inhibitors such as ketoconazole, ciclosporin, itraconazole or dronedarone (GmbH, 2020). The blood concentration of dabigatran is increased by about 2.4-fold and 2.3-fold when dabigatran is co-administered with dronedarone (GmbH, 2020), which may increase the bleeding risk (Reilly et al., 2014; Sinigoj et al., 2015). Our study also showed the increased risk of major bleedings when concurrent use of apixaban with SNRIs compared with apixaban alone. Not only serve as CYP3A4 substrates, apixaban are also metabolized by P-gp (Steffel et al., 2018). Concomitant use of rifampin, an inducer of CYP3A4 and P-gp, decreases blood concentration of apixaban by 50% (Squibb, 2021). However, the data for the inhibitors of CYP3A4 and P-gp remain insufficient. Given that both duloxetine and venlafaxine inhibit P-gp activities, it is reasonable to propose that concurrently use of apixaban and SNRIs may increase the concentration of apixaban as well as bleeding risk. More pharmacokinetics and clinical studies regarding interactions between individual DOACs and antidepressants will be warranted to confirm these findings.

An increased risk of ICH was observed in AF patients with DOACs concurrently taking TeCAs. Although metabolized by CYP3A4 (Rotzinger et al., 1998), trazodone only weakly inhibits the activity of CYP3A4 (Caccia, 2007). Mirtazapine may not affect the activity of CYP3A4 as well (Anttila and Leinonen, 2001). In cell culture, trazodone may induce expression of intestinal P-gp, leading to the reduced absorption of co-administered oral medications (Störmer et al., 2001). Mirtazapine is not a substrate of P-gp (O’Brien et al., 2012). Administration with mirtazapine to P-gp knockout mice is not affect its concentration in brain (O’Brien et al., 2012). Further studies will be warranted to clarify the mechanisms of TeCAs in the increased ICH risk of these patients.

Tricyclic antidepressants do not appear to increase bleeding risk when used alone (de Abajo et al., 1999; Schalekamp et al., 2008). However, they could potentially increase the bleeding risk with warfarin through their inhibitory effects on warfarin metabolism. In rats, amitriptyline demonstrated a dose-dependent increase in prothrombin time, which correlated with an increase in the plasma half-life of warfarin (Loomis and Racz, 1980). However, a case–control study determined that the administration of TCAs and coumarin did not confer a higher risk of gastrointestinal bleeding compared with coumarin alone (Schalekamp et al., 2008). Our results indicate that, as a class, TCA is not associated with any major bleeding in the patients with AF taking DOACs.

This study has some limitations. Misclassification bias may become an issue in the nationwide registration studies, although large data sets could potentially overcome this problem. Studies to evaluate the validity of diagnosis codes in the NHI research database also showed modest to high sensitivity and positive predictive values in epilepsy (Chen et al., 2012), ischemic stroke (Hsieh et al., 2015), hypertension, diabetes, hyperlipidemia and atrial fibrillation (Sung et al., 2016). Laboratory data regarding renal or liver function were not available in the dataset. Thus, the results may have been biased by unmeasured confounders. However, these biases were minimized by incorporating an extensive list of covariates that represent proximal indicators representing the severity of renal or liver disease and other clinical factors typically considered by clinicians prescribing DOACs. Other drugs, such as lipid-lowering drugs, antiplatelets or non-steroid anti-inflammatory drugs, may influence bleeding risk, although an extensive adjustment in medications has been performed in our model. Around 30% of patients with hypertension in our population did not receive pharmacological treatment and may increase the risk of major bleeding, while analysis of short time interval with person-quarter with adjustment for anti-hypertensives may reduce this bias. The high prevalence of *Helicobacter pylori* infection and peptic ulcer diseases in Han-Chinese population may undermine the association between gastrointestinal hemorrhage and selected medications (Nagy et al., 2016). Furthermore, DOAC and antidepressant dosage, duration and compliance were not considered in the model. The associations between bleeding risk and short-term use of medications were not identified as well. These potential misclassifications of drug exposure likely biased the results toward negative findings, and the risk of bleeding may have been underestimated. Additionally, detecting an increased bleeding risk associated with drug–drug interactions between individual DOACs and antidepressants when the number of bleeding events is small may be difficult. Exposures of multiple antidepressants may also affect the results, although the number is small (0.61%). Finally, ethnic differences in bleeding risk with oral anticoagulant therapy may also limit the generalizability of our findings to other populations (Chan et al., 2016).

## Conclusion

In conclusion, we found concurrent use of bupropion with DOACs in patients with non-valvular AF was associated with an increased risk of major bleeding, particularly in gastrointestinal hemorrhage. The combination of an DOAC and SSRIs, particularly in paroxetine, and TeCAs was associated with a higher risk of ICH. SSRIs increased the risk of ICH in the dabigatran-treated patients with AF. The combination of apixaban and SNRIs was associated with a higher risk of major bleeding. These drug–drug interactions should be carefully considered when prescribing DOACs in adult patients.

## Data Availability Statement

The original contributions presented in the study are included in the article/Supplementary Material, further inquiries can be directed to the corresponding author/s.

## Ethics Statement

The studies involving human participants were reviewed and approved by the Institutional Review Board of Chang Gung Memorial Hospital, Linkou, Taiwan (ethical license no: 201901357B0). The ethics committee waived the requirement of written informed consent for participation.

## Author Contributions

K-HC contributed to the conceptualization, carried out the funding acquisition, investigated the data, performed the methodology, wrote the original draft, and reviewed and edited the manuscript. C-MC contributed to the conceptualization, investigated the data, performed the methodology, wrote the original draft, and reviewed and edited the manuscript. C-LW investigated the data, performed the methodology, and wrote the original draft. H-TT carried out the data curation, formal analysis, software, and project administration. Y-TH carried out the data curation and formal analysis, and performed the methodology. H-CW and C-HC investigated the data and performed the methodology. S-HC contributed to the conceptualization, carried out the funding acquisition, investigated the data, performed the methodology, supervised the data, carried out the resources, and wrote, reviewed, and edited the manuscript. All authors contributed to the article and approved the submitted version.

## Conflict of Interest

The authors declare that the research was conducted in the absence of any commercial or financial relationships that could be construed as a potential conflict of interest.

## Publisher’s Note

All claims expressed in this article are solely those of the authors and do not necessarily represent those of their affiliated organizations, or those of the publisher, the editors and the reviewers. Any product that may be evaluated in this article, or claim that may be made by its manufacturer, is not guaranteed or endorsed by the publisher.

## Glossary

AF, atrial fibrillation; ARR, adjusted rate ratio; CI, confidence interval; CYP3A4, cytochrome P450 3A4; DOAC, direct oral anticoagulant; ICH, intracerebral hemorrhage; NHI, Taiwan National Health Insurance; P-gp, permeability glycoprotein; SNRI, selective serotonin–norepinephrine reuptake inhibitor; SSRI, selective serotonin reuptake inhibitors; TCA, tricyclic antidepressant; TeCA, tetracyclic antidepressant.
