# Supplementary material for: Major Bleeding Risk in Patients With Non-valvular Atrial Fibrillation Concurrently Taking Direct Oral Anticoagulants and Antidepressants
Source: Front Aging Neurosci. 2022 Feb 4;14:791285. doi: 10.3389/fnagi.2022.791285 (PMC8855103; doi:10.3389/fnagi.2022.791285)
Supplement: Supplementary file 1 [file Data_Sheet_1.docx]

**Supplementary Table 1.** Definitions and codes of major bleeding and negative control outcomes.

|  | ICD-9-CM | | ICD-10 | |
| --- | --- | --- | --- | --- |
| Intracranial | 430, 431, 432.0, 432.1, 432.9 | | I60, I61, I62 | |
| Gastrointestinal | 530.7, 531, 531.2, 531.4, 531.6, 532, 532.2, 532.4, 532.6, 533, 533.2, 533.4, 533.6, 534, 534.2, 534.4, 534.6, 569.3, 535.01, 53.11, 535.21, 535.31, 535.41, 535.51, 535.61, 535.71, 537.71, 537.83, 537.84, 562.02, 562.03, 562.12, 562.13, 569.85, 578 | | K22.6, K25–K28, K29.01, K29.21, K29.31, K29.41, K29.51, K29.61, K29.71, K29.81, K29.91, K31.811, K31.82, K52.81, K55.21, K56.60, K57.01, K57.11, K57.13, K57.21, K57.31, K57.33, K57.81, K57.91, K57.93, K62.5, K92.0, K92.1, K92.2 | |
| Other sites (intraspinal, intraocular, retroperitoneal, intra-articular, pericardial, or intramuscular bleeding) | 336.1, 363.6, 372.72, 376.32, 377.42, 379.23, 593.81, 866.01, 866.02, 866.11, 866.12, 719.1, 729.92, 423.0, 772.5 | | G95.11, G95.19, H05.23, H11.3, H31.3, H43.1, H47.02, I31.2, M25.0, N28.0, P54.4, S31.001A, S37.011A, S37.012A, S37.019A, S37.021A, S37.022A, S37.029A, S37.031A, A37.032A, S37.039A, S37.041A, S37.042A, S37.049A, S37.051A, S37.052A, S37.059A | |
| Acute pancreatitis | | 577.0 | | K85 |
| Intestinal obstruction †without mention of hernia | | 560 | | K56 |
| Acute appendicitis | | 540, 541 | | K35 |

**Supplementary Table 2.** Definitions and codes of atrial fibrillation and covariates.

|  | ICD-9-CM | ICD-10 |
| --- | --- | --- |
| Atrial fibrillation | 427.31 | I48.0, I48.2, I48.91 |
| Hypertension | 401, 402 | I10–I16 |
| Myocardial infarction | 410, 412 | I21, I22, I25.2 |
| Congestive heart failure | 398.91, 402.01, 402.11, 402.91, 404.01, 404.03, 404.11, 404.13, 404.91, 404.93, 425.4–425.9, 428 | I09.9, I11.0, I13.0, I13.2, I25.5, I42.0, I42.5–I42.9, I43, I50, I29.0 |
| Percutaneous coronary intervention* | 33076A, 33076B, 33077A, 33077B, 33078A, 33078B | |
| Coronary artery bypass surgery* | 68023A, 68023B, 68024A, 68024B, 68025A, 68025B | |
| Peripheral vascular disease | 093.0, 437.3, 440, 441, 443.1–443.9, 557.1, 557.9, V43.4 | I70, I71, I73.1, I73.8, I73.9, I77.1, I79.0, I79.2, K55.1, K55.8, K55.9, Z95.8, Z95.9 |
| Cerebrovascular disease | 362.34, 430–438 | G45, G46, H34.0, I60–I69 |
| Ischemic stroke | 433–434, 436, 852, 853 | I67.89, I63–I64, G458–G459, S01.90XA, S06.4X0A–S06.4X9A, S06.5X0A–S06.5X9A, S06.6X0A–S06.6X9A, S06.340A–S06.349A, S06.350A–S06.359A, S06.360A–S06.369A |
| Transient ischemic attack | 435 | G45 |
| Hemiplegia or paraplegia | 334.1, 342, 343, 344.0–344.6, 344.9 | G04.1, G11.4, G80.1, G80.2, G81, G82, G83.0–G83.4, G83.9 |
| Dementia | 290, 294.1, 331.2 | F00–F03, F05.1, G30, G31.1 |
| Epilepsy | 345 | G40 |
| Diabetes mellitus | 250 | E10.0, E10.1, E10.9, E11.0, E11.1, E11.9 |
| Diabetes with complications | 250.4–250.7 | E10.2–E10.5, E10.7, E11.2–E11.5, E11.7, E12.2–E12.5, E12.7, E13.2–E13.5, E13.7, E14.2–E14.5, E14.7 |
| Chronic kidney disease | 580-589 | I12, I13, N00–N05, N07, N11, N14, N17, N18, N19, Q61 |
| Chronic pulmonary disease | 416.8, 416.9, 490–505, 506.4, 508.1, 508.8 | I27.8, I27.9, J40–J47, J60–J67, J68.4, J70.1, J70.3 |
| Chronic obstructive pulmonary disease | 490, 491.0, 491.1, 491.20, 491.21, 491.22, 491.8, 492.0, 492.8, 494, 496 | J40, J41.0, J41.1, J41.8, J42, J43.0, J43.1, J43.2, J43.8, J43.9, J44.0, J44.1, J44.9, J47.0, J47.1, J47.9 |
| Peptic ulcer disease | 531–534 | K25–K28 |
| Mild liver disease | 070.22, 070.23, 070.32, 070.33, 070.44, 070.54, 070.6, 070.9, 570, 571, 573.3, 573.4, 573.8, 573.9 | B18, K70.0–K70.3, K70.9, K71.3–K71.5, K71.7, K73, K74, K76.0, K76.2–K76.4, K76.8, K76.9, Z94.4 |
| Moderate or severe liver disease | 456.0–456.2, 572.2–572.8 | I85.0, I85.9, I86.4, I98.2, K70.4, K71.1, K72.1, K72.9, K76.5, K76.6, K76.7 |
| Any malignancy, including lymphoma and leukemia, except malignant neoplasm of skin | 140–172, 174-195.8, 200–208, 238.6 | C00–C26, C30–C34, C37–C41, C43, C45–C58, C60–C76, C81–C85, C88, C90–C97 |
| Metastatic solid tumor | 196–199 | C77–C80 |
| Human immunodeficiency virus infection | 042–044 | B20–B22, B24 |
| Anemia | 280–285 | D46.1, D46.4, D50–D64 |
| Rheumatic disease | 446.5, 710.0–710.4, 714.0–714.2, 714.8, 725 | M05, M06, M31.5, M32–M34, M35.1, M35.3, M36.0 |

*Percutaneous coronary intervention / coronary artery bypass surgery is procedure code.

**Supplementary Table 3.** Demographic analysis of patients taking direct oral anticoagulants (DOACs).

|  | DOAC users (n = 98 863) |
| --- | --- |
| Age (years) | 74.89 ± 10.32 |
| Male (%) | 55 610 (56.25%) |
| Age (years) | 74.89 ± 10.32 |
| Male (%) | 55 610 (56.25%) |
| CHAD_2_DS_2_-VASc score | 4.69 ± 1.83 |
| Anemia (%) | 15 520 (15.7%) |
| Acute pancreatitis (%) | 1639 (1.66%) |
| Acute appendicitis (%) | 1601 (1.62%) |
| Cancer (%) | 14 064 (14.23%) |
| Metastatic solid tumor (%) | 1334 (1.35%) |
| Cardiovascular diseases |  |
| Hypertension (%) | 86 688 (87.68%) |
| Myocardial infarction (%) | 6507 (6.58%) |
| Congestive heart failure (%) | 51 300 (51.89%) |
| Peripheral vascular disease (%) | 13 896 (14.06%) |
| Peripheral arterial occlusive disease (%) | 2677 (2.71%) |
| Percutaneous coronary intervention (%) | 8660 (8.76%) |
| Coronary artery bypass surgery (%) | 1121 (1.13%) |
| Chronic kidney disease (%) | 26 568 (26.87%) |
| Gastrointestinal and hepatic diseases |  |
| Peptic ulcer disease (%) | 56 455 (57.10%) |
| Mild liver disease (%) | 36 580 (37.00%) |
| Moderate or severe liver disease (%) | 334 (0.34%) |
| Human immunodeficiency virus infection (%) | 25 (0.03%) |
| Intestinal obstruction †without mention of hernia (%) | 5147 (5.21%) |
| Metabolic disease |  |
| Diabetes Mellitus (%) | 41 790 (42.27%) |
| Diabetes with complications (%) | 14 745 (14.91%) |
| Neurological diseases |  |
| Cerebral vascular disease (%) | 50 422 (51.00%) |
| Ischemic stroke (%) | 37 610 (38.04%) |
| Transient ischemic attack (%) | 12 915 (13.06%) |
| Hemiplegia and paraplegia (%) | 5096 (5.15%) |
| Dementia (%) | 11 427 (11.56%) |
| Epilepsy (%) | 2793 (2.83%) |
| Pulmonary disease |  |
| Chronic pulmonary disease (%) | 52 619 (53.22%) |
| Chronic obstructive pulmonary disease (%) | 47 585 (48.13%) |
| Residence |  |
| Urban (%) | 52 055 (52.65%) |
| Suburban (%) | 32 384 (32.76%) |
| Rural (%) | 13 761 (13.92%) |
| Unknown (%) | 663 (0.67%) |
| Occupation |  |
| Dependents of the insured individuals (%) | 38 834 (39.28%) |
| Civil servants, teachers, military personnel and veterans (%) | 11 812 (11.95%) |
| Nonmanual workers and professionals (%) | 5706 (5.77%) |
| Manual workers (%) | 31 636 (32.00%) |
| Others (%) | 10 719 (10.84%) |
| Income |  |
| Quantile 1 (%) | 28 810 (29.14%) |
| Quantile 2 (%) | 1475 (1.49%) |
| Quantile 3 (%) | 41 130 (41.6%) |
| Quantile 4 (%) | 9738 (9.85%) |
| Quantile 5 (%) | 17 492 (17.69%) |
| Unknown (%) | 218 (0.22%) |

**Supplementary Table 4.** Medications during follow-up.

| Medications | DOAC users (n = 98 863) |
| --- | --- |
| Antibiotics and antifungal drugs | 2800 (2.83%) |
| Erythromycin (%) | 2277 (2.3%) |
| Itraconazole (%) | 64 (0.06) |
| Ketoconazole (%) | 163 (0.16%) |
| Rifampin (%) | 311 (0.31%) |
| Agomelatine | 653 (3.33%) |
| Anticoagulants | 98 863 (100%) |
| Apixaban | 20 825 (21.06%) |
| Dabigatran | 42 779 (43.27%) |
| Edoxaban | 10 517 (10.64%) |
| Rivaroxaban | 55 587 (56.23%) |
| Antidepressants | 19 632 (19.86%) |
| Amitriptyline | 3183 (16.21%) |
| Bupropion | 534 (2.72%) |
| Citalopram | 3695 (18.82%) |
| Duloxetine | 1536 (7.82%) |
| Escitalopram | 3442 (17.53%) |
| Fluoxetine | 934 (4.76%) |
| Imipramine | 6177 (31.45) |
| Melitracen | 3183 (16.21%) |
| Mirtazapine | 1656 (8.43%) |
| Paroxetine | 875 (4.46%) |
| Sertraline | 2751 (14.01%) |
| Trazodone | 4922 (25.06%) |
| Venlafaxine | 626 (3.19%) |
| Antiepileptics | 4928 (4.98%) |
| Carbamazepine | 189 (0.19%) |
| Gabapentin | 758 (0.77%) |
| Lamotrigine | 42 (0.04%) |
| Levetiracetam | 1081 (1.09%) |
| Oxcarbazepine | 322 (0.33%) |
| Phenobarbital | 814 (0.82%) |
| Phenytoin | 651 (0.66%) |
| Pregabalin | 408 (0.41%) |
| Topiramate | 138 (0.14%) |
| Valproate | 1289 (1.3%) |
| Antihypertensives | 59 324 (60.1%) |
| Bisoprolol (%) | 33 396 (33.78%) |
| Diltiazem (%) | 15 284 (15.46%) |
| Irbesartan (%) | 4478 (4.53%) |
| Labetalol (%) | 1187 (1.2%) |
| Losartan (%) | 8784 (8.89%) |
| Metoprolol (%) | 1131 (1.14%) |
| Olmesartan (%) | 6055 (6.12%) |
| Propranolol (%) | 8026 (8.12%) |
| Antiplatelets | 24 186 (24.46%) |
| Aspirin (%) | 13 873 (14.03%) |
| Cilostazol | 1792 (1.81%) |
| Clopidogrel (%) | 6063 (6.13%) |
| Dipyridamole | 3404 (3.44%) |
| Pentoxifylline | 3724 (3.77%) |
| Ticagrelor (%) | 376 (0.38%) |
| Ticlopidine (%) | 808 (0.82%) |
| Bisphosphate (%) | 602 (0.61%) |
| Cardiovascular drugs | 36 505 (36.92%) |
| Amiodarone (%) | 20 328 (20.56%) |
| Digoxin (%) | 16 933 (17.13%) |
| Dronedarone (%) | 2907 (2.94%) |
| Cyclosporine (%) | 49 (0.05%) |
| Glucocorticoid (%) | 7987 (8.08%) |
| Insulin (%) | 6236 (6.31%) |
| Lipid lowering drugs | 19 172 (19.39%) |
| Atorvastatin (%) | 12 654 (12.8%) |
| Ezetimibe (%) | 82 (0.08%) |
| Fluvastatin (%) | 1436 (1.45%) |
| Pravastatin (%) | 1600 (1.62%) |
| Pitavastatin (%) | 3726 (3.77%) |
| Non-steroid anti-inflammatory drugs (%) | 19 180 (19.4%) |

| **Supplementary Table 5**. Covariate balance before and after inverse probability of treatment weighting using propensity score among patients with AF taking direct oral anticoagulants (DOACs) with or without concurrent selective serotonin–norepinephrine reuptake inhibitors (SNRIs). | | | | | | | |
| --- | --- | --- | --- | --- | --- | --- | --- |
|  | Propensity score weighting | | | | | | |
|  | Before | | |  | After | | |
| Characteristic | Person quarters with DOAC use | Person quarters with DOAC and SNRI use | ASMD |  | Person quarters with DOAC use | Person quarters with DOAC and SNRI use | ASMD |
| Age (years) | 75.75 ± 9.82 | 75.31 ± 9.43 | 0.0461 |  | 75.61 ± 0.93 | 75.31 ± 9.43 | 0.0443 |
| Male | 408430 (58.44%) | 3153 (47.47%) | 0.2012 |  | 3155 (47.36%) | 3153 (47.47%) | <0.0001 |
| CHAD2DS2-VASc score | 3.43 ± 1.46 | 3.76 ± 1.5 | 0.2217 |  | 3.76 ± 0.15 | 3.76 ± 1.5 | 0.0042 |
| Anemia | 20958 (3%) | 271 (4.08%) | 0.0585 |  | 237 (3.55%) | 271 (4.08%) | 0.0274 |
| Acute pancreatitis | 1057 (0.15%) | 12 (0.18%) | 0.0072 |  | 16 (0.23%) | 12 (0.18%) | 0.0118 |
| Acute appendicitis | 373 (0.05%) | 0 (0%) | 0.0327 |  | 4 (0.06%) | 0 (0%) | 0.0335 |
| Cancer | 35693 (5.11%) | 454 (6.84%) | 0.0730 |  | 370 (5.56%) | 454 (6.84%) | 0.0529 |
| Metastatic solid tumor | 2367 (0.34%) | 32 (0.48%) | 0.0224 |  | 28 (0.42%) | 32 (0.48%) | 0.0088 |
| Cardiovascular diseases |  |  |  |  |  |  |  |
| Hypertension | 409312 (58.57%) | 4044 (60.89%) | 0.0473 |  | 4055 (60.88%) | 4044 (60.89%) | 0.0002 |
| Myocardial infarction | 13861 (1.98%) | 96 (1.45%) | 0.0415 |  | 96 (1.45%) | 96 (1.45%) | 0.0003 |
| Congestive heart failure | 182016 (26.04%) | 1471 (22.15%) | 0.0912 |  | 1479 (22.2%) | 1471 (22.15%) | 0.0013 |
| Peripheral vascular disease | 15812 (2.26%) | 190 (2.86%) | 0.0379 |  | 192 (2.88%) | 190 (2.86%) | 0.0011 |
| Percutaneous coronary intervention | 3054 (0.44%) | 36 (0.54%) | 0.0150 |  | 36 (0.54%) | 36 (0.54%) | 0.0001 |
| Chronic kidney disease | 73661 (10.54%) | 725 (10.92%) | 0.0121 |  | 731 (10.98%) | 725 (10.92%) | 0.0020 |
| Gastrointestinal and hepatic diseases |  |  |  |  |  |  |  |
| Peptic ulcer disease | 69755 (9.98%) | 979 (14.74%) | 0.1450 |  | 990(14.86%) | 979(14.74%) | 0.0033 |
| Mild liver disease | 35515(5.08%) | 401(6.04%) | 0.0417 |  | 411(6.16%) | 401(6.04%) | 0.0053 |
| Intestinal obstruction without mention of hernia | 5150(0.74%) | 86(1.29%) | 0.0557 |  | 63(0.95%) | 86(1.29%) | 0.0332 |
| Metabolic disease |  |  |  |  |  |  |  |
| Diabetes Mellitus | 151461 (21.67%) | 1882 (28.33%) | 0.1543 |  | 1893 (28.42%) | 1882 (28.33%) | 0.0018 |
| Diabetes with complications | 48029 (6.87%) | 898 (13.52%) | 0.2210 |  | 626 (9.4%) | 898 (13.52%) | 0.1295 |
| Neurological diseases |  |  |  |  |  |  |  |
| Cerebral vascular disease | 209185 (29.93%) | 2925 (44.04%) | 0.2954 |  | 2934 (44.04%) | 2925 (44.04%) | 0.0001 |
| Ischemic stroke | 141790 (20.29%) | 2004 (30.17%) | 0.2290 |  | 2010 (30.17%) | 2004 (30.17%) | <0.0001 |
| Transient ischemic attack | 19495 (2.79%) | 239 (3.6%) | 0.0460 |  | 240(3.61%) | 239(3.6%) | 0.0005 |
| Hemiplegia and paraplegia | 12768(1.83%) | 263(3.96%) | 0.1275 |  | 186(2.79%) | 263(3.96%) | 0.0646 |
| Dementia | 43333(6.2%) | 1164(17.52%) | 0.3557 |  | 505(7.58%) | 1164(17.52%) | 0.3038 |
| Epilepsy | 9203(1.32%) | 167(2.51%) | 0.0874 |  | 163(2.44%) | 167(2.51%) | 0.1295 |
| Medications |  |  |  |  |  |  |  |
| Aspirin | 164326 (23.51%) | 1857 (27.96%) | 0.1018 |  | 1873 (28.11%) | 1857 (27.96%) | 0.0034 |
| Clopidogrel | 65022 (9.3%) | 792 (11.92%) | 0.0851 |  | 799 (11.99%) | 792 (11.92%) | 0.0021 |
| Ticlopidine | 8804 (1.26%) | 169 (2.54%) | 0.0942 |  | 170 (2.55%) | 169 (2.54%) | 0.0004 |
| Ticagrelor | 6633 (0.95%) | 60 (0.9%) | 0.0048 |  | 61 (0.91%) | 60 (0.9%) | 0.0010 |
| Bisoprolol | 294142 (42.09%) | 2657 (40%) | 0.0424 |  | 2677 (40.19%) | 2657 (40%) | 0.0038 |
| Amiodarone | 179651 (25.71%) | 1923 (28.95%) | 0.0729 |  | 1935 (29.05%) | 1923 (28.95%) | 0.0022 |
| Digoxin | 167106 (23.91%) | 1572 (23.67%) | 0.0057 |  | 1586 (23.81%) | 1572 (23.67%) | 0.0033 |
| Diltiazem | 157299 (22.51%) | 1802 (27.13%) | 0.1072 |  | 1817 (27.28%) | 1802 (27.13%) | 0.0034 |
| Metoprolol | 12536 (1.79%) | 207 (3.12%) | 0.0856 |  | 207 (3.1%) | 207 (3.12%) | 0.0009 |
| Labetalol | 26062 (3.73%) | 462 (6.96%) | 0.1439 |  | 470 (7.06%) | 462 (6.96%) | 0.0039 |
| Propranolol | 92980 (13.3%) | 1342 (20.2%) | 0.1856 |  | 1353 (20.31%) | 1342 (20.2%) | 0.0027 |
| Dronedarone | 30489 (4.36%) | 283 (4.26%) | 0.0050 |  | 283 (4.25%) | 283 (4.26%) | 0.0007 |
| Itraconazole | 1744 (0.25%) | 22 (0.33%) | 0.0152 |  | 22 (0.34%) | 22 (0.33%) | 0.0009 |
| Ketoconazole | 5230 (0.75%) | 83 (1.25%) | 0.0504 |  | 83 (1.24%) | 83 (1.25%) | 0.0008 |
| Erythromycin | 52312 (7.49%) | 419 (6.31%) | 0.0465 |  | 419 (6.29%) | 419 (6.31%) | 0.0006 |
| Atorvastatin | 130979 (18.74%) | 1460 (21.98%) | 0.0805 |  | 1467 (22.02%) | 1460 (21.98%) | 0.0010 |
| Rifampin | 3762 (0.54%) | 33 (0.5%) | 0.0058 |  | 33 (0.5%) | 33 (0.5%) | 0.0003 |
| Bisphosphonates | 8163 (1.17%) | 141 (2.12%) | 0.0751 |  | 144 (2.16%) | 141 (2.12%) | 0.0023 |
| Fluvastatin | 18024 (2.58%) | 219 (3.3%) | 0.0425 |  | 220 (3.31%) | 219 (3.3%) | 0.0004 |
| Pravastatin | 20147 (2.88%) | 213 (3.21%) | 0.0189 |  | 213 (3.2%) | 213 (3.21%) | 0.0004 |
| Pitavastatin | 44101 (6.31%) | 458 (6.9%) | 0.0236 |  | 460 (6.91%) | 458 (6.9%) | 0.0004 |
| Ezetimib | 1718 (0.25%) | 31 (0.47%) | 0.0371 |  | 31 (0.47%) | 31 (0.47%) | 0.0005 |
| Insulin | 64362 (9.21%) | 1111 (16.73%) | 0.2252 |  | 1131 (16.98%) | 1111 (16.73%) | 0.0069 |
| Irbesartan | 51698 (7.4%) | 404 (6.08%) | 0.0525 |  | 405 (6.09%) | 404 (6.08%) | 0.0002 |
| Losartan | 92333 (13.21%) | 997 (15.01%) | 0.0517 |  | 1005 (15.09%) | 997 (15.01%) | 0.0021 |
| Olmesartan | 67707 (9.69%) | 716 (10.78%) | 0.0360 |  | 721 (10.82%) | 716 (10.78%) | 0.0014 |
| Glucocorticoids | 125019 (17.89%) | 1465 (22.06%) | 0.1044 |  | 1482 (22.24%) | 1465 (22.06%) | 0.0045 |
| Non-steroid anti-inflammatory drugs | 289198 (41.38%) | 3131 (47.14%) | 0.1161 |  | 3144 (47.2%) | 3131 (47.14%) | 0.0013 |
| Carbamazepine | 2779 (0.4%) | 46 (0.69%) | 0.0401 |  | 48 (0.72%) | 46 (0.69%) | 0.0037 |
| Gabapentin | 13487 (1.93%) | 429 (6.46%) | 0.2274 |  | 445 (6.69%) | 429 (6.46%) | 0.0092 |
| Lamotrigine | 558 (0.08%) | 47 (0.71%) | 0.1004 |  | 49 (0.74%) | 47 (0.71%) | 0.0034 |
| Levetiracetam | 11756 (1.68%) | 157 (2.36%) | 0.0484 |  | 161 (2.41%) | 157 (2.36%) | 0.0033 |
| Oxcarbazepine | 4444 (0.64%) | 220 (3.31%) | 0.1933 |  | 233 (3.5%) | 220 (3.31%) | 0.0105 |
| Phenobarbital | 19138 (2.74%) | 220 (3.31%) | 0.0335 |  | 219 (3.29%) | 220 (3.31%) | 0.0013 |
| Phenytoin | 7958 (1.14%) | 137 (2.06%) | 0.0737 |  | 141 (2.12%) | 137 (2.06%) | 0.0040 |
| Pregabalin | 7537 (1.08%) | 415 (6.25%) | 0.2778 |  | 433 (6.5%) | 415 (6.25%) | 0.0102 |
| Topiramate | 1716 (0.25%) | 61 (0.92%) | 0.0885 |  | 65 (0.97%) | 61 (0.92%) | 0.0052 |
| Valproate | 14573 (2.09%) | 356 (5.36%) | 0.1736 |  | 360 (5.4%) | 356 (5.36%) | 0.0018 |
| Cilostazol | 20624 (2.95%) | 333 (5.01%) | 0.1056 |  | 339 (5.09%) | 333 (5.01%) | 0.0036 |
| Dipyridamole | 45821 (6.56%) | 536 (8.07%) | 0.0582 |  | 543 (8.15%) | 536 (8.07%) | 0.0031 |
| Pentoxifylline | 53396 (7.64%) | 803 (12.09%) | 0.1496 |  | 817 (12.27%) | 803 (12.09%) | 0.0055 |
| ASMD: Absolute standardized mean difference. | | | | | | | |

| **Supplementary Table 6**. Covariate balance before and after inverse probability of treatment weighting using propensity score among patients with AF taking direct oral anticoagulants (DOACs) with or without concurrent selective serotonin reuptake inhibitors (SSRIs) | | | | | | | |
| --- | --- | --- | --- | --- | --- | --- | --- |
|  | Propensity score weighting | | | | | | |
|  | Before | | |  | After | | |
| Characteristic | Person quarters with DOAC use | Person quarters with DOAC and SSRI use | ASMD |  | Person quarters with DOAC use | Person quarters with DOAC and SSRI use | ASMD |
| Age (years) | 75.75 ± 9.82 | 76.45 ± 9.64 | 0.0745 |  | 76.47 ± 1.86 | 76.45 ± 9.64 | 0.0027 |
| Male | 399692 (58.76%) | 11891 (46.92%) | 0.2214 |  | 11885 (46.84%) | 11891 (46.92%) | <0.0001 |
| CHAD2DS2-VASc score | 3.42 ± 1.46 | 3.85 ± 1.52 | 0.2885 |  | 3.85 ± 0.29 | 3.85 ± 1.52 | 0.0003 |
| Anemia | 20280 (2.98%) | 949 (3.74%) | 0.0423 |  | 845 (3.33%) | 949 (3.74%) | 0.0224 |
| Acute pancreatitis | 1034 (0.15%) | 35 (0.14%) | 0.0037 |  | 46 (0.18%) | 35 (0.14%) | 0.0108 |
| Acute appendicitis | 355 (0.05%) | 18 (0.07%) | 0.0076 |  | 13 (0.05%) | 18 (0.07%) | 0.0081 |
| Cancer | 34791 (5.11%) | 1356 (5.35%) | 0.0106 |  | 1312 (5.17%) | 1356 (5.35%) | 0.0080 |
| Metastatic solid tumor | 2310 (0.34%) | 89 (0.35%) | 0.0020 |  | 87 (0.34%) | 89 (0.35%) | 0.0012 |
| Cardiovascular diseases |  |  |  |  |  |  |  |
| Hypertension | 398141 (58.53%) | 15215 (60.03%) | 0.0305 |  | 15233 (60.04%) | 15215 (60.03%) | 0.0001 |
| Myocardial infarction | 13513 (1.99%) | 444 (1.75%) | 0.0173 |  | 443 (1.75%) | 444 (1.75%) | 0.0003 |
| Congestive heart failure | 177862 (26.15%) | 5625 (22.19%) | 0.0925 |  | 5631 (22.19%) | 5625 (22.19%) | <0.0001 |
| Peripheral vascular disease | 15437 (2.27%) | 565 (2.23%) | 0.0027 |  | 567 (2.24%) | 565 (2.23%) | 0.0004 |
| Percutaneous coronary intervention | 3002 (0.44%) | 88 (0.35%) | 0.0150 |  | 88 (0.35%) | 88 (0.35%) | 0.0001 |
| Chronic kidney disease | 71876 (10.57%) | 2510 (9.9%) | 0.0219 |  | 2516 (9.92%) | 2510 (9.9%) | 0.0005 |
| Gastrointestinal and hepatic diseases |  |  |  |  |  |  |  |
| Peptic ulcer disease | 67608 (9.94%) | 3126 (12.33%) | 0.0762 |  | 3140 (12.38%) | 3126 (12.33%) | 0.0013 |
| Mild liver disease | 34580 (5.08%) | 1336 (5.27%) | 0.0085 |  | 1340 (5.28%) | 1336 (5.27%) | 0.0005 |
| Intestinal obstruction without mention of hernia | 4942 (0.73%) | 294 (1.16%) | 0.0449 |  | 217 (0.86%) | 294 (1.16%) | 0.0303 |
| Metabolic disease |  |  |  |  |  |  |  |
| Diabetes Mellitus | 147800 (21.73%) | 5543 (21.87%) | 0.0034 |  | 5556 (21.9%) | 5543 (21.87%) | 0.0006 |
| Diabetes with complications | 47236 (6.94%) | 1691 (6.67%) | 0.0108 |  | 1728 (6.81%) | 1691 (6.67%) | 0.0055 |
| Neurological diseases |  |  |  |  |  |  |  |
| Cerebral vascular disease | 200098 (29.42%) | 12012 (47.4%) | 0.3761 |  | 12053 (47.5%) | 12012 (47.4%) | 0.0022 |
| Ischemic stroke | 135127 (19.87%) | 8667 (34.2%) | 0.3270 |  | 8699 (34.28%) | 8667 (34.2%) | 0.0018 |
| Transient ischemic attack | 18813 (2.77%) | 921 (3.63%) | 0.0493 |  | 923 (3.64%) | 921 (3.63%) | 0.0002 |
| Hemiplegia and paraplegia | 11761 (1.73%) | 1270 (5.01%) | 0.1826 |  | 764 (3.01%) | 1270 (5.01%) | 0.1021 |
| Dementia | 39047 (5.74%) | 5450 (21.5%) | 0.4722 |  | 1922 (7.57%) | 5450 (21.5%) | 0.4031 |
| Epilepsy | 8761 (1.29%) | 609 (2.4%) | 0.0829 |  | 710 (2.8%) | 609 (2.4%) | 0.0055 |
| Medications |  |  |  |  |  |  |  |
| Aspirin | 158467 (23.3%) | 7716 (30.45%) | 0.1618 |  | 7737 (30.49%) | 7716 (30.45%) | 0.0010 |
| Clopidogrel | 62703 (9.22%) | 3111 (12.28%) | 0.0988 |  | 3121 (12.3%) | 3111 (12.28%) | 0.0008 |
| Ticlopidine | 8417 (1.24%) | 556 (2.19%) | 0.0737 |  | 564 (2.22%) | 556 (2.19%) | 0.0020 |
| Ticagrelor | 6432 (0.95%) | 261 (1.03%) | 0.0085 |  | 261 (1.03%) | 261 (1.03%) | 0.0001 |
| Bisoprolol | 286309 (42.09%) | 10490 (41.39%) | 0.0143 |  | 10515 (41.44%) | 10490 (41.39%) | 0.0010 |
| Amiodarone | 174211 (25.61%) | 7363 (29.05%) | 0.0772 |  | 7399 (29.16%) | 7363 (29.05%) | 0.0024 |
| Digoxin | 162664 (23.91%) | 6014 (23.73%) | 0.0044 |  | 6035 (23.79%) | 6014 (23.73%) | 0.0013 |
| Diltiazem | 152623 (22.44%) | 6478 (25.56%) | 0.0731 |  | 6507 (25.65%) | 6478 (25.56%) | 0.0020 |
| Metoprolol | 12242 (1.8%) | 501 (1.98%) | 0.0130 |  | 499 (1.97%) | 501 (1.98%) | 0.0007 |
| Labetalol | 24737 (3.64%) | 1787 (7.05%) | 0.1522 |  | 1804 (7.11%) | 1787 (7.05%) | 0.0023 |
| Propranolol | 89722 (13.19%) | 4600 (18.15%) | 0.1367 |  | 4628 (18.24%) | 4600 (18.15%) | 0.0023 |
| Dronedarone | 29485 (4.33%) | 1287 (5.08%) | 0.0351 |  | 1293 (5.1%) | 1287 (5.08%) | 0.0008 |
| Itraconazole | 1668 (0.25%) | 98 (0.39%) | 0.0252 |  | 98 (0.38%) | 98 (0.39%) | 0.0003 |
| Ketoconazole | 5097 (0.75%) | 216 (0.85%) | 0.0115 |  | 219 (0.86%) | 216 (0.85%) | 0.0013 |
| Erythromycin | 50709 (7.46%) | 2022 (7.98%) | 0.0196 |  | 2036 (8.02%) | 2022 (7.98%) | 0.0017 |
| Atorvastatin | 127010 (18.67%) | 5429 (21.42%) | 0.0687 |  | 5440 (21.44%) | 5429 (21.42%) | 0.0005 |
| Rifampin | 3699 (0.54%) | 96 (0.38%) | 0.0244 |  | 96 (0.38%) | 96 (0.38%) | 0.0002 |
| Bisphosphonates | 7930 (1.17%) | 374 (1.48%) | 0.0271 |  | 378 (1.49%) | 374 (1.48%) | 0.0013 |
| Fluvastatin | 17545 (2.58%) | 698 (2.75%) | 0.0108 |  | 700 (2.76%) | 698 (2.75%) | 0.0004 |
| Pravastatin | 19607 (2.88%) | 753 (2.97%) | 0.0052 |  | 761 (3%) | 753 (2.97%) | 0.0016 |
| Pitavastatin | 43006 (6.32%) | 1553 (6.13%) | 0.0081 |  | 1558 (6.14%) | 1553 (6.13%) | 0.0005 |
| Ezetimib | 1710 (0.25%) | 39 (0.15%) | 0.0217 |  | 39 (0.15%) | 39 (0.15%) | <0.0001 |
| Insulin | 62783 (9.23%) | 2690 (10.61%) | 0.0463 |  | 2704 (10.66%) | 2690 (10.61%) | 0.0014 |
| Irbesartan | 50184 (7.38%) | 1918 (7.57%) | 0.0072 |  | 1922 (7.57%) | 1918 (7.57%) | 0.0003 |
| Losartan | 89943 (13.22%) | 3387 (13.36%) | 0.0041 |  | 3392 (13.37%) | 3387 (13.36%) | 0.0002 |
| Olmesartan | 65670 (9.65%) | 2753 (10.86%) | 0.0398 |  | 2754 (10.85%) | 2753 (10.86%) | 0.0003 |
| Glucocorticoids | 121942 (17.93%) | 4542 (17.92%) | 0.0002 |  | 4568 (18.01%) | 4542 (17.92%) | 0.0022 |
| Non-steroid anti-inflammatory drugs | 281182 (41.34%) | 11147 (43.98%) | 0.0535 |  | 11175 (44.04%) | 11147 (43.98%) | 0.0012 |
| Carbamazepine | 2702 (0.4%) | 123 (0.49%) | 0.0133 |  | 124 (0.49%) | 123 (0.49%) | 0.0005 |
| Gabapentin | 13219 (1.94%) | 697 (2.75%) | 0.0533 |  | 704 (2.78%) | 697 (2.75%) | 0.0015 |
| Lamotrigine | 568 (0.08%) | 37 (0.15%) | 0.0185 |  | 38 (0.15%) | 37 (0.15%) | 0.0014 |
| Levetiracetam | 10931 (1.61%) | 982 (3.87%) | 0.1392 |  | 1000 (3.94%) | 982 (3.87%) | 0.0035 |
| Oxcarbazepine | 4351 (0.64%) | 313 (1.24%) | 0.0618 |  | 320 (1.26%) | 313 (1.24%) | 0.0023 |
| Phenobarbital | 18757 (2.76%) | 601 (2.37%) | 0.0244 |  | 603 (2.38%) | 601 (2.37%) | 0.0004 |
| Phenytoin | 7554 (1.11%) | 541 (2.13%) | 0.0811 |  | 548 (2.16%) | 541 (2.13%) | 0.0017 |
| Pregabalin | 7532 (1.11%) | 420 (1.66%) | 0.0471 |  | 423 (1.67%) | 420 (1.66%) | 0.0007 |
| Topiramate | 1677 (0.25%) | 100 (0.39%) | 0.0262 |  | 101 (0.4%) | 100 (0.39%) | 0.0008 |
| Valproate | 13421 (1.97%) | 1508 (5.95%) | 0.2050 |  | 1535 (6.05%) | 1508 (5.95%) | 0.0043 |
| Cilostazol | 20178 (2.97%) | 779 (3.07%) | 0.0063 |  | 782 (3.08%) | 779 (3.07%) | 0.0005 |
| Dipyridamole | 44391 (6.53%) | 1966 (7.76%) | 0.0478 |  | 1974 (7.78%) | 1966 (7.76%) | 0.0009 |
| Pentoxifylline | 51948 (7.64%) | 2251 (8.88%) | 0.0452 |  | 2258 (8.9%) | 2251 (8.88%) | 0.0006 |

ASMD: Absolute standardized mean difference.

| **Supplementary Table 7**. Covariate balance before and after inverse probability of treatment weighting using propensity score among patients with AF taking direct oral anticoagulants (DOACs) with or without concurrent tricyclic antidepressants (TCAs). | | | | | | | |
| --- | --- | --- | --- | --- | --- | --- | --- |
|  | Propensity score weighting | | | | | | |
|  | Before | | |  | After | | |
| Characteristic | Person quarters with DOAC use | Person quarters with DOAC and TCA use | ASMD |  | Person quarters with DOAC use | Person quarters with DOAC and TCA use | ASMD |
| Age (years) | 75.75 ± 9.82 | 76.4 ± 8.92 | 0.0745 |  | 76.41 ± 1.76 | 76.4 ± 8.92 | 0.0027 |
| Male | 398288 (58.53%) | 13295 (53%) | 0.2214 |  | 13307 (52.97%) | 13295 (53%) | <0.0001 |
| CHAD2DS2-VASc score | 3.42 ± 1.46 | 3.67 ± 1.43 | 0.2885 |  | 3.68 ± 0.28 | 3.67 ± 1.43 | 0.0003 |
| Anemia | 20377 (2.99%) | 852 (3.4%) | 0.0229 |  | 847 (3.37%) | 852 (3.4%) | 0.0013 |
| Acute pancreatitis | 1030 (0.15%) | 39 (0.16%) | 0.0011 |  | 45 (0.18%) | 39 (0.16%) | 0.0057 |
| Acute appendicitis | 361 (0.05%) | 12 (0.05%) | 0.0023 |  | 14 (0.06%) | 12 (0.05%) | 0.0037 |
| Cancer | 34514 (5.07%) | 1633 (6.51%) | 0.0616 |  | 1359 (5.41%) | 1633 (6.51%) | 0.0464 |
| Metastatic solid tumor | 2315 (0.34%) | 84 (0.33%) | 0.0009 |  | 93 (0.37%) | 84 (0.33%) | 0.0058 |
| Cardiovascular diseases |  |  |  |  |  |  |  |
| Hypertension | 397760 (58.46%) | 15596 (62.18%) | 0.0305 |  | 15630 (62.22%) | 15596 (62.18%) | 0.0001 |
| Myocardial infarction | 13545 (1.99%) | 412 (1.64%) | 0.0173 |  | 412 (1.64%) | 412 (1.64%) | 0.0003 |
| Congestive heart failure | 177608 (26.1%) | 5879 (23.44%) | 0.0925 |  | 5882 (23.42%) | 5879 (23.44%) | <0.0001 |
| Peripheral vascular disease | 15159 (2.23%) | 843 (3.36%) | 0.0027 |  | 852 (3.39%) | 843 (3.36%) | 0.0004 |
| Percutaneous coronary intervention | 2986 (0.44%) | 104 (0.41%) | 0.0150 |  | 104 (0.41%) | 104 (0.41%) | 0.0001 |
| Chronic kidney disease | 71294 (10.48%) | 3092 (12.33%) | 0.0219 |  | 3108 (12.37%) | 3092 (12.33%) | 0.0005 |
| Gastrointestinal and hepatic diseases |  |  |  |  |  |  |  |
| Peptic ulcer disease | 67422 (9.91%) | 3312 (13.2%) | 0.0762 |  | 3332 (13.26%) | 3312 (13.2%) | 0.0013 |
| Mild liver disease | 34416 (5.06%) | 1500 (5.98%) | 0.0085 |  | 1505 (5.99%) | 1500 (5.98%) | 0.0005 |
| Intestinal obstruction without mention of hernia | 4954 (0.73%) | 282 (1.12%) | 0.0414 |  | 212 (0.84%) | 282 (1.12%) | 0.0284 |
| Metabolic disease |  |  |  |  |  |  |  |
| Diabetes Mellitus | 147136 (21.62%) | 6207 (24.75%) | 0.0034 |  | 6223 (24.77%) | 6207 (24.75%) | 0.0006 |
| Diabetes with complications | 46443 (6.83%) | 2484 (9.9%) | 0.1113 |  | 1995 (7.94%) | 2484 (9.9%) | 0.0688 |
| Neurological diseases |  |  |  |  |  |  |  |
| Cerebral vascular disease | 203070 (29.84%) | 9040 (36.04%) | 0.3761 |  | 9074 (36.12%) | 9040 (36.04%) | 0.0022 |
| Ischemic stroke | 137673 (20.23%) | 6121 (24.4%) | 0.3270 |  | 6143 (24.45%) | 6121 (24.4%) | 0.0018 |
| Transient ischemic attack | 18701 (2.75%) | 1033 (4.12%) | 0.0493 |  | 1046 (4.16%) | 1033 (4.12%) | 0.0002 |
| Hemiplegia and paraplegia | 12357 (1.82%) | 674 (2.69%) | 0.0587 |  | 528 (2.1%) | 674 (2.69%) | 0.0382 |
| Dementia | 42149 (6.19%) | 2348 (9.36%) | 0.1184 |  | 1706 (6.79%) | 2348 (9.36%) | 0.0943 |
| Epilepsy | 9014 (1.32%) | 356 (1.42%) | 0.0081 |  | 358 (1.42%) | 356 (1.42%) | 0.0005 |
| Medications |  |  |  |  |  |  |  |
| Aspirin100 | 159617 (23.46%) | 6566 (26.18%) | 0.1618 |  | 6596 (26.26%) | 6566 (26.18%) | 0.0010 |
| Clopidogrel | 63277 (9.3%) | 2537 (10.11%) | 0.0988 |  | 2557 (10.18%) | 2537 (10.11%) | 0.0008 |
| Ticlopidine | 8585 (1.26%) | 388 (1.55%) | 0.0737 |  | 387 (1.54%) | 388 (1.55%) | 0.0020 |
| Ticagrelor | 6439 (0.95%) | 254 (1.01%) | 0.0085 |  | 255 (1.01%) | 254 (1.01%) | 0.0001 |
| Bisoprolol | 286522 (42.11%) | 10277 (40.97%) | 0.0143 |  | 10306 (41.03%) | 10277 (40.97%) | 0.0010 |
| Amiodarone | 174946 (25.71%) | 6628 (26.42%) | 0.0772 |  | 6648 (26.47%) | 6628 (26.42%) | 0.0024 |
| Digoxin | 162695 (23.91%) | 5983 (23.85%) | 0.0044 |  | 5996 (23.87%) | 5983 (23.85%) | 0.0013 |
| Diltiazem | 152996 (22.48%) | 6105 (24.34%) | 0.0731 |  | 6123 (24.38%) | 6105 (24.34%) | 0.0020 |
| Metoprolol | 12255 (1.8%) | 488 (1.95%) | 0.0130 |  | 489 (1.95%) | 488 (1.95%) | 0.0007 |
| Labetalol | 25355 (3.73%) | 1169 (4.66%) | 0.1522 |  | 1189 (4.73%) | 1169 (4.66%) | 0.0023 |
| Propranolol | 89655 (13.18%) | 4667 (18.61%) | 0.1367 |  | 4693 (18.68%) | 4667 (18.61%) | 0.0023 |
| Dronedarone | 29555 (4.34%) | 1217 (4.85%) | 0.0351 |  | 1221 (4.86%) | 1217 (4.85%) | 0.0008 |
| Itraconazole | 1675 (0.25%) | 91 (0.36%) | 0.0252 |  | 93 (0.37%) | 91 (0.36%) | 0.0003 |
| Ketoconazole | 5115 (0.75%) | 198 (0.79%) | 0.0115 |  | 199 (0.79%) | 198 (0.79%) | 0.0013 |
| Erythromycin | 50528 (7.43%) | 2203 (8.78%) | 0.0196 |  | 2212 (8.81%) | 2203 (8.78%) | 0.0017 |
| Atorvastatin | 127413 (18.73%) | 5026 (20.04%) | 0.0687 |  | 5036 (20.05%) | 5026 (20.04%) | 0.0005 |
| Rifampin | 3655 (0.54%) | 140 (0.56%) | 0.0244 |  | 140 (0.56%) | 140 (0.56%) | 0.0002 |
| Bisphosphonates | 7814 (1.15%) | 490 (1.95%) | 0.0271 |  | 499 (1.99%) | 490 (1.95%) | 0.0013 |
| Fluvastatin | 17603 (2.59%) | 640 (2.55%) | 0.0108 |  | 636 (2.53%) | 640 (2.55%) | 0.0004 |
| Pravastatin | 19619 (2.88%) | 741 (2.95%) | 0.0052 |  | 742 (2.96%) | 741 (2.95%) | 0.0016 |
| Pitavastatin | 42889 (6.3%) | 1670 (6.66%) | 0.0081 |  | 1678 (6.68%) | 1670 (6.66%) | 0.0005 |
| Ezetimib | 1680 (0.25%) | 69 (0.28%) | 0.0217 |  | 68 (0.27%) | 69 (0.28%) | <0.0001 |
| Insulin | 62782 (9.23%) | 2691 (10.73%) | 0.0463 |  | 2706 (10.77%) | 2691 (10.73%) | 0.0014 |
| Irbesartan | 50074 (7.36%) | 2028 (8.09%) | 0.0072 |  | 2028 (8.08%) | 2028 (8.09%) | 0.0003 |
| Losartan | 90026 (13.23%) | 3304 (13.17%) | 0.0041 |  | 3317 (13.2%) | 3304 (13.17%) | 0.0002 |
| Olmesartan | 65935 (9.69%) | 2488 (9.92%) | 0.0398 |  | 2506 (9.98%) | 2488 (9.92%) | 0.0003 |
| Glucocorticoids | 121329 (17.83%) | 5155 (20.55%) | 0.0002 |  | 5188 (20.65%) | 5155 (20.55%) | 0.0022 |
| Non-steroid anti-inflammatory drugs | 280458 (41.22%) | 11871 (47.33%) | 0.0535 |  | 11902 (47.38%) | 11871 (47.33%) | 0.0012 |
| Carbamazepine | 2549 (0.37%) | 276 (1.1%) | 0.0133 |  | 299 (1.19%) | 276 (1.1%) | 0.0005 |
| Gabapentin | 12778 (1.88%) | 1138 (4.54%) | 0.0533 |  | 1171 (4.66%) | 1138 (4.54%) | 0.0015 |
| Lamotrigine | 583 (0.09%) | 22 (0.09%) | 0.0185 |  | 22 (0.09%) | 22 (0.09%) | 0.0014 |
| Levetiracetam | 11582 (1.7%) | 331 (1.32%) | 0.1392 |  | 335 (1.33%) | 331 (1.32%) | 0.0035 |
| Oxcarbazepine | 4214 (0.62%) | 450 (1.79%) | 0.0618 |  | 479 (1.91%) | 450 (1.79%) | 0.0023 |
| Phenobarbital | 18537 (2.72%) | 821 (3.27%) | 0.0244 |  | 824 (3.28%) | 821 (3.27%) | 0.0004 |
| Phenytoin | 7762 (1.14%) | 333 (1.33%) | 0.0811 |  | 337 (1.34%) | 333 (1.33%) | 0.0017 |
| Pregabalin | 7114 (1.05%) | 838 (3.34%) | 0.0471 |  | 870 (3.47%) | 838 (3.34%) | 0.0007 |
| Topiramate | 1627 (0.24%) | 150 (0.6%) | 0.0262 |  | 156 (0.62%) | 150 (0.6%) | 0.0008 |
| Valproate | 14325 (2.11%) | 604 (2.41%) | 0.2050 |  | 607 (2.42%) | 604 (2.41%) | 0.0043 |
| Cilostazol | 19837 (2.92%) | 1120 (4.47%) | 0.0063 |  | 1137 (4.53%) | 1120 (4.47%) | 0.0005 |
| Dipyridamole | 44094 (6.48%) | 2263 (9.02%) | 0.0478 |  | 2283 (9.09%) | 2263 (9.02%) | 0.0009 |
| Pentoxifylline | 51540 (7.57%) | 2659 (10.6%) | 0.0452 |  | 2685 (10.69%) | 2659 (10.6%) | 0.0006 |

ASMD: Absolute standardized mean difference.

| **Supplementary Table 8**. Covariate balance before and after inverse probability of treatment weighting using propensity score among patients with AF taking direct oral anticoagulants (DOACs) with or without concurrent tetracyclic antidepressants (TeCAs). | | | | | | | |
| --- | --- | --- | --- | --- | --- | --- | --- |
|  | Propensity score weighting | | | | | | |
|  | Before | | |  | After | | |
| Characteristic | Person quarters with DOAC use | Person quarters with DOAC and TeCA use | ASMD |  | Person quarters with DOAC use | Person quarters with DOAC and TeCA use | ASMD |
| Age (years) | 75.75 ± 9.82 | 76.99 ± 9.36 | 0.1326 |  | 76.91 ± 1.57 | 76.99 ± 9.36 | 0.0118 |
| Male | 401629 (58.52%) | 9954 (51.9%) | 0.1206 |  | 9952 (51.85%) | 9954 (51.9%) | <0.0001 |
| CHAD2DS2-VASc score | 3.42 ± 1.46 | 3.8 ± 1.51 | 0.2563 |  | 3.81 ± 0.25 | 3.8 ± 1.51 | 0.0029 |
| Anemia | 20443 (2.98%) | 786 (4.1%) | 0.0606 |  | 682 (3.55%) | 786 (4.1%) | 0.0285 |
| Acute pancreatitis | 1027 (0.15%) | 42 (0.22%) | 0.0162 |  | 37 (0.19%) | 42 (0.22%) | 0.0055 |
| Acute appendicitis | 365 (0.05%) | 8 (0.04%) | 0.0053 |  | 11 (0.06%) | 8 (0.04%) | 0.0069 |
| Cancer | 34858 (5.08%) | 1289 (6.72%) | 0.0697 |  | 1046 (5.45%) | 1289 (6.72%) | 0.0531 |
| Metastatic solid tumor | 2316 (0.34%) | 83 (0.43%) | 0.0154 |  | 68 (0.35%) | 83 (0.43%) | 0.0125 |
| Cardiovascular diseases |  |  |  |  |  |  |  |
| Hypertension | 401513 (58.5%) | 11843 (61.75%) | 0.0664 |  | 11854 (61.76%) | 11843 (61.75%) | 0.0003 |
| Myocardial infarction | 13550 (1.97%) | 407 (2.12%) | 0.0104 |  | 409 (2.13%) | 407 (2.12%) | 0.0005 |
| Congestive heart failure | 178894 (26.06%) | 4593 (23.95%) | 0.0489 |  | 4594 (23.94%) | 4593 (23.95%) | 0.0002 |
| Peripheral vascular disease | 15348 (2.24%) | 654 (3.41%) | 0.0709 |  | 657 (3.43%) | 654 (3.41%) | 0.0009 |
| Percutaneous coronary intervention | 3012 (0.44%) | 78 (0.41%) | 0.0050 |  | 78 (0.41%) | 78 (0.41%) | 0.0001 |
| Chronic kidney disease | 72019 (10.49%) | 2367 (12.34%) | 0.0581 |  | 2374 (12.37%) | 2367 (12.34%) | 0.0008 |
| Gastrointestinal and hepatic diseases |  |  |  |  |  |  |  |
| Peptic ulcer disease | 68177 (9.93%) | 2557 (13.33%) | 0.1062 |  | 2564 (13.36%) | 2557 (13.33%) | 0.0009 |
| Mild liver disease | 34740 (5.06%) | 1176 (6.13%) | 0.0466 |  | 1179 (6.14%) | 1176 (6.13%) | 0.0005 |
| Intestinal obstruction without mention of hernia | 4986 (0.73%) | 250 (1.3%) | 0.0576 |  | 175 (0.91%) | 250 (1.3%) | 0.0373 |
| Metabolic disease |  |  |  |  |  |  |  |
| Diabetes Mellitus | 148609 (21.65%) | 4734 (24.68%) | 0.0719 |  | 4736 (24.68%) | 4734 (24.68%) | 0.0002 |
| Diabetes with complications | 47331 (6.9%) | 1596 (8.32%) | 0.0538 |  | 1490 (7.76%) | 1596 (8.32%) | 0.0205 |
| Neurological diseases |  |  |  |  |  |  |  |
| Cerebral vascular disease | 204067 (29.73%) | 8043 (41.94%) | 0.2566 |  | 8063 (42.01%) | 8043 (41.94%) | 0.0015 |
| Ischemic stroke | 138153 (20.13%) | 5641 (29.41%) | 0.2163 |  | 5656 (29.47%) | 5641 (29.41%) | 0.0012 |
| Transient ischemic attack | 19079 (2.78%) | 655 (3.42%) | 0.0367 |  | 658 (3.43%) | 655 (3.42%) | 0.0006 |
| Hemiplegia and paraplegia | 12255 (1.79%) | 776 (4.05%) | 0.1347 |  | 498 (2.6%) | 776 (4.05%) | 0.0810 |
| Dementia | 41024 (5.98%) | 3473 (18.11%) | 0.3794 |  | 1455 (7.58%) | 3473 (18.11%) | 0.3185 |
| Epilepsy | 9018 (1.31%) | 352 (1.84%) | 0.0419 |  | 391 (2.04%) | 352 (1.84%) | 0.0147 |
| Medications |  |  |  |  |  |  |  |
| Aspirin | 160710 (23.42%) | 5473 (28.54%) | 0.1170 |  | 5487 (28.59%) | 5473 (28.54%) | 0.0012 |
| Clopidogrel | 63539 (9.26%) | 2275 (11.86%) | 0.0848 |  | 2281 (11.89%) | 2275 (11.86%) | 0.0007 |
| Ticlopidine | 8629 (1.26%) | 344 (1.79%) | 0.0438 |  | 346 (1.8%) | 344 (1.79%) | 0.0006 |
| Ticagrelor | 6530 (0.95%) | 163 (0.85%) | 0.0107 |  | 163 (0.85%) | 163 (0.85%) | <0.0001 |
| Bisoprolol | 289132 (42.13%) | 7667 (39.98%) | 0.0437 |  | 7673 (39.98%) | 7667 (39.98%) | 0.0001 |
| Amiodarone | 175857 (25.62%) | 5717 (29.81%) | 0.0936 |  | 5732 (29.87%) | 5717 (29.81%) | 0.0013 |
| Digoxin | 164285 (23.94%) | 4393 (22.91%) | 0.0243 |  | 4398 (22.91%) | 4393 (22.91%) | 0.0002 |
| Diltiazem | 154692 (22.54%) | 4409 (22.99%) | 0.0107 |  | 4423 (23.04%) | 4409 (22.99%) | 0.0013 |
| Metoprolol | 12326 (1.8%) | 417 (2.17%) | 0.0271 |  | 415 (2.16%) | 417 (2.17%) | 0.0007 |
| Labetalol | 25599 (3.73%) | 925 (4.82%) | 0.0541 |  | 927 (4.83%) | 925 (4.82%) | 0.0004 |
| Propranolol | 90834 (13.23%) | 3488 (18.19%) | 0.1364 |  | 3501 (18.24%) | 3488 (18.19%) | 0.0014 |
| Dronedarone | 29951 (4.36%) | 821 (4.28%) | 0.0041 |  | 820 (4.28%) | 821 (4.28%) | 0.0003 |
| Itraconazole | 1687 (0.25%) | 79 (0.41%) | 0.0290 |  | 80 (0.42%) | 79 (0.41%) | 0.0005 |
| Ketoconazole | 5112 (0.74%) | 201 (1.05%) | 0.0322 |  | 203 (1.06%) | 201 (1.05%) | 0.0009 |
| Erythromycin | 51011 (7.43%) | 1720 (8.97%) | 0.0560 |  | 1726 (8.99%) | 1720 (8.97%) | 0.0008 |
| Atorvastatin | 128571 (18.73%) | 3868 (20.17%) | 0.0363 |  | 3875 (20.19%) | 3868 (20.17%) | 0.0005 |
| Rifampin | 3673 (0.54%) | 122 (0.64%) | 0.0132 |  | 122 (0.63%) | 122 (0.64%) | 0.0002 |
| Bisphosphonates | 7844 (1.14%) | 460 (2.4%) | 0.0953 |  | 465 (2.42%) | 460 (2.4%) | 0.0015 |
| Fluvastatin | 17720 (2.58%) | 523 (2.73%) | 0.0090 |  | 524 (2.73%) | 523 (2.73%) | 0.0001 |
| Pravastatin | 19841 (2.89%) | 519 (2.71%) | 0.0112 |  | 521 (2.71%) | 519 (2.71%) | 0.0005 |
| Pitavastatin | 43589 (6.35%) | 970 (5.06%) | 0.0558 |  | 972 (5.07%) | 970 (5.06%) | 0.0004 |
| Ezetimib | 1676 (0.24%) | 73 (0.38%) | 0.0244 |  | 73 (0.38%) | 73 (0.38%) | 0.0003 |
| Insulin | 63185 (9.21%) | 2288 (11.93%) | 0.0887 |  | 2295 (11.96%) | 2288 (11.93%) | 0.0009 |
| Irbesartan | 50841 (7.41%) | 1261 (6.57%) | 0.0327 |  | 1260 (6.56%) | 1261 (6.57%) | 0.0004 |
| Losartan | 90917 (13.25%) | 2413 (12.58%) | 0.0198 |  | 2413 (12.57%) | 2413 (12.58%) | 0.0002 |
| Olmesartan | 66273 (9.66%) | 2150 (11.21%) | 0.0509 |  | 2147 (11.19%) | 2150 (11.21%) | 0.0007 |
| Glucocorticoids | 122618 (17.87%) | 3866 (20.16%) | 0.0584 |  | 3875 (20.19%) | 3866 (20.16%) | 0.0008 |
| Non-steroid anti-inflammatory drugs | 283682 (41.33%) | 8647 (45.09%) | 0.0758 |  | 8661 (45.13%) | 8647 (45.09%) | 0.0009 |
| Carbamazepine | 2712 (0.4%) | 113 (0.59%) | 0.0277 |  | 114 (0.59%) | 113 (0.59%) | 0.0004 |
| Gabapentin | 13206 (1.92%) | 710 (3.7%) | 0.1077 |  | 718 (3.74%) | 710 (3.7%) | 0.0020 |
| Lamotrigine | 573 (0.08%) | 32 (0.17%) | 0.0236 |  | 33 (0.17%) | 32 (0.17%) | 0.0009 |
| Levetiracetam | 11476 (1.67%) | 437 (2.28%) | 0.0436 |  | 439 (2.29%) | 437 (2.28%) | 0.0006 |
| Oxcarbazepine | 4480 (0.65%) | 184 (0.96%) | 0.0343 |  | 186 (0.97%) | 184 (0.96%) | 0.0008 |
| Phenobarbital | 18825 (2.74%) | 533 (2.78%) | 0.0022 |  | 535 (2.79%) | 533 (2.78%) | 0.0006 |
| Phenytoin | 7766 (1.13%) | 329 (1.72%) | 0.0493 |  | 332 (1.73%) | 329 (1.72%) | 0.0010 |
| Pregabalin | 7605 (1.11%) | 347 (1.81%) | 0.0585 |  | 351 (1.83%) | 347 (1.81%) | 0.0015 |
| Topiramate | 1696 (0.25%) | 81 (0.42%) | 0.0303 |  | 82 (0.43%) | 81 (0.42%) | 0.0011 |
| Valproate | 14089 (2.05%) | 840 (4.38%) | 0.1322 |  | 847 (4.41%) | 840 (4.38%) | 0.0016 |
| Cilostazol | 20297 (2.96%) | 660 (3.44%) | 0.0275 |  | 663 (3.45%) | 660 (3.44%) | 0.0006 |
| Dipyridamole | 44737 (6.52%) | 1620 (8.45%) | 0.0733 |  | 1625 (8.47%) | 1620 (8.45%) | 0.0007 |
| Pentoxifylline | 52304 (7.62%) | 1895 (9.88%) | 0.0800 |  | 1903 (9.92%) | 1895 (9.88%) | 0.0012 |

ASMD: Absolute standardized mean difference.

| **Supplementary Table 9**. Covariate balance before and after inverse probability of treatment weighting using propensity score among patients with AF taking direct oral anticoagulants (DOACs) with or without other antidepressants. | | | | | | | |
| --- | --- | --- | --- | --- | --- | --- | --- |
|  | Propensity score weighting | | | | | | |
|  | Before | | |  | After | | |
| Characteristic | Person quarters with DOAC use | Person quarters with DOAC and other antidepressants | ASMD |  | Person quarters with DOAC use | Person quarters with DOAC and other antidepressants | ASMD |
| Age (years) | 75.75 ± 9.82 | 75.93 ± 9.73 | 0.0185 |  | 76.12 ± 0.68 | 75.93 ± 9.73 | 0.0276 |
| Male | 409760 (58.37%) | 1823 (51.92%) | 0.1407 |  | 1823 (51.93%) | 1823 (51.92%) | <0.0001 |
| CHAD2DS2-VASc score | 3.43 ± 1.46 | 3.65 ± 1.47 | 0.1508 |  | 3.65 ± 0.11 | 3.65 ± 1.47 | 0.0043 |
| Anemia | 21073 (3%) | 156 (4.44%) | 0.0762 |  | 118.8713 (3.39%) | 156 (4.44%) | 0.0546 |
| Acute pancreatitis | 1065 (0.15%) | 4 (0.11%) | 0.0104 |  | 6.3053 (0.18%) | 4 (0.11%) | 0.0171 |
| Acute appendicitis | 373 (0%) | 0 (0%) | 0.0326 |  | 2 (0%) | 0 (0%) | 0.0325 |
| Cancer | 35940 (5.12%) | 207 (5.9%) | 0.0340 |  | 190.0423 (5.41%) | 207 (5.9%) | 0.0209 |
| Metastatic solid tumor | 2378 (0.34%) | 21 (0.6%) | 0.0380 |  | 12.6031 (0.36%) | 21 (0.6%) | 0.0347 |
| Cardiovascular diseases |  |  |  |  |  |  |  |
| Hypertension | 411242 (58.58%) | 2114 (60.21%) | 0.0332 |  | 2115 (60.22%) | 2114 (60.21%) | 0.0001 |
| Myocardial infarction | 13897 (1.98%) | 60 (1.71%) | 0.0201 |  | 60 (1.71%) | 60 (1.71%) | <0.0001 |
| Congestive heart failure | 182696 (26.02%) | 791 (22.53%) | 0.0816 |  | 791 (22.52%) | 791 (22.53%) | 0.0003 |
| Peripheral vascular disease | 15922 (2.27%) | 80 (2.28%) | 0.0007 |  | 80 (2.28%) | 80 (2.28%) | 0.0001 |
| Percutaneous coronary intervention | 3080 (0.44%) | 10 (0.28%) | 0.0256 |  | 10 (0.29%) | 10 (0.28%) | <0.0001 |
| Chronic kidney disease | 73976 (10.54%) | 410 (11.68%) | 0.0363 |  | 410 (11.68%) | 410 (11.68%) | 0.0002 |
| Gastrointestinal and hepatic diseases |  |  |  |  |  |  |  |
| Peptic ulcer disease | 70255 (10.01%) | 479 (13.64%) | 0.1128 |  | 479 (13.65%) | 479 (13.64%) | 0.0002 |
| Mild liver disease | 35671 (5.08%) | 245 (6.98%) | 0.0797 |  | 245 (6.98%) | 245 (6.98%) | 0.0001 |
| Intestinal obstruction without mention of hernia | 5176 (0.74%) | 60 (1.71%) | 0.0885 |  | 30.1024 (0.86%) | 60 (1.71%) | 0.0757 |
| Metabolic disease |  |  |  |  |  |  |  |
| Diabetes Mellitus | 152537 (21.73%) | 806 (22.96%) | 0.0295 |  | 806 (22.95%) | 806 (22.96%) | 0.0001 |
| Diabetes with complications | 48622 (6.93%) | 305 (8.69%) | 0.0657 |  | 256.8468 (7.31%) | 305 (8.69%) | 0.0506 |
| Neurological diseases |  |  |  |  |  |  |  |
| Cerebral vascular disease | 210768 (30.02%) | 1342 (38.22%) | 0.1736 |  | 1343 (38.25%) | 1342 (38.22%) | 0.0006 |
| Ischemic stroke | 142859 (20.35%) | 935 (26.63%) | 0.1486 |  | 936 (26.65%) | 935 (26.63%) | 0.0003 |
| Transient ischemic attack | 19602 (2.79%) | 132 (3.76%) | 0.0544 |  | 132 (3.76%) | 132 (3.76%) | <0.0001 |
| Hemiplegia and paraplegia | 12890 (1.84%) | 141 (4.02%) | 0.1296 |  | 84.4836 (2.41%) | 141 (4.02%) | 0.0914 |
| Dementia | 43531 (6.2%) | 966 (27.51%) | 0.5939 |  | 257.8696 (7.34%) | 966 (27.51%) | 0.5515 |
| Epilepsy | 9308 (1.33%) | 62 (1.77%) | 0.0357 |  | 75.0097 (2.14%) | 62 (1.77%) | 0.0268 |
| Medications |  |  |  |  |  |  |  |
| Aspirin100 | 165207 (23.53%) | 976 (27.8%) | 0.0978 |  | 976 (27.8%) | 976 (27.8%) | 0.0001 |
| Clopidogrel | 65447 (9.32%) | 367 (10.45%) | 0.0379 |  | 367 (10.46%) | 367 (10.45%) | 0.0004 |
| Ticlopidine | 8909 (1.27%) | 64 (1.82%) | 0.0449 |  | 64 (1.82%) | 64 (1.82%) | 0.0001 |
| Ticagrelor | 6669 (0.95%) | 24 (0.68%) | 0.0296 |  | 24 (0.68%) | 24 (0.68%) | <.0001 |
| Bisoprolol | 295391 (42.08%) | 1408 (40.1%) | 0.0402 |  | 1408 (40.11%) | 1408 (40.1%) | 0.0001 |
| Amiodarone | 180495 (25.71%) | 1079 (30.73%) | 0.1117 |  | 1080 (30.76%) | 1079 (30.73%) | 0.0006 |
| Digoxin | 167875 (23.91%) | 803 (22.87%) | 0.0246 |  | 803 (22.88%) | 803 (22.87%) | 0.0003 |
| Diltiazem | 158110 (22.52%) | 991 (28.23%) | 0.1313 |  | 992 (28.24%) | 991 (28.23%) | 0.0002 |
| Metoprolol | 12660 (1.8%) | 83 (2.36%) | 0.0393 |  | 83 (2.36%) | 83 (2.36%) | <.0001 |
| Labetalol | 26327 (3.75%) | 197 (5.61%) | 0.0882 |  | 198 (5.63%) | 197 (5.61%) | 0.0008 |
| Propranolol | 93520 (13.32%) | 802 (22.84%) | 0.2493 |  | 802 (22.85%) | 802 (22.84%) | 0.0001 |
| Dronedarone | 30566 (4.35%) | 206 (5.87%) | 0.0688 |  | 205 (5.84%) | 206 (5.87%) | 0.0011 |
| Itraconazole | 1750 (0.25%) | 16 (0.46%) | 0.0348 |  | 16 (0.46%) | 16 (0.46%) | 0.0003 |
| Ketoconazole | 5272 (0.75%) | 41 (1.17%) | 0.0428 |  | 41 (1.16%) | 41 (1.17%) | 0.0004 |
| Erythromycin | 52377 (7.46%) | 354 (10.08%) | 0.0928 |  | 354 (10.09%) | 354 (10.08%) | 0.0002 |
| Atorvastatin | 131733 (18.77%) | 706 (20.11%) | 0.0339 |  | 707 (20.12%) | 706 (20.11%) | 0.0004 |
| Rifampin | 3785 (0.54%) | 10 (0.28%) | 0.0397 |  | 10 (0.28%) | 10 (0.28%) | <.0001 |
| Bisphosphonates | 8242 (1.17%) | 62 (1.77%) | 0.0492 |  | 62 (1.77%) | 62 (1.77%) | 0.0002 |
| Fluvastatin | 18132 (2.58%) | 111 (3.16%) | 0.0346 |  | 111 (3.16%) | 111 (3.16%) | 0.0002 |
| Pravastatin | 20224 (2.88%) | 136 (3.87%) | 0.0550 |  | 136 (3.87%) | 136 (3.87%) | 0.0001 |
| Pitavastatin | 44326 (6.31%) | 233 (6.64%) | 0.0131 |  | 233 (6.65%) | 233 (6.64%) | 0.0004 |
| Ezetimib | 1744 (0.25%) | 5 (0.14%) | 0.0240 |  | 5 (0.14%) | 5 (0.14%) | <.0001 |
| Insulin | 65089 (9.27%) | 384 (10.94%) | 0.0553 |  | 384 (10.94%) | 384 (10.94%) | 0.0002 |
| Irbesartan | 51800 (7.38%) | 302 (8.6%) | 0.0451 |  | 302 (8.61%) | 302 (8.6%) | 0.0001 |
| Losartan | 92833 (13.22%) | 497 (14.16%) | 0.0271 |  | 497 (14.15%) | 497 (14.16%) | 0.0001 |
| Olmesartan | 67949 (9.68%) | 474 (13.5%) | 0.1196 |  | 474 (13.5%) | 474 (13.5%) | 0.0001 |
| Glucocorticoids | 125736 (17.91%) | 748 (21.3%) | 0.0856 |  | 749 (21.32%) | 748 (21.3%) | 0.0004 |
| Non-steroid anti-inflammatory drugs | 290678 (41.41%) | 1651 (47.02%) | 0.1133 |  | 1652 (47.04%) | 1651 (47.02%) | 0.0004 |
| Carbamazepine | 2806 (0.4%) | 19 (0.54%) | 0.0207 |  | 19 (0.54%) | 19 (0.54%) | 0.0003 |
| Gabapentin | 13791 (1.96%) | 125 (3.56%) | 0.0975 |  | 125 (3.57%) | 125 (3.56%) | 0.0004 |
| Lamotrigine | 587 (0.08%) | 18 (0.51%) | 0.0788 |  | 19 (0.53%) | 18 (0.51%) | 0.0020 |
| Levetiracetam | 11834 (1.69%) | 79 (2.25%) | 0.0406 |  | 79 (2.25%) | 79 (2.25%) | 0.0003 |
| Oxcarbazepine | 4639 (0.66%) | 25 (0.71%) | 0.0062 |  | 25 (0.72%) | 25 (0.71%) | 0.0004 |
| Phenobarbital | 19291 (2.75%) | 67 (1.91%) | 0.0557 |  | 67 (1.91%) | 67 (1.91%) | 0.0001 |
| Phenytoin | 8064 (1.15%) | 31 (0.88%) | 0.0265 |  | 31 (0.88%) | 31 (0.88%) | 0.0002 |
| Pregabalin | 7902 (1.13%) | 50 (1.42%) | 0.0266 |  | 50 (1.43%) | 50 (1.42%) | 0.0004 |
| Topiramate | 1747 (0.25%) | 30 (0.85%) | 0.0818 |  | 30 (0.86%) | 30 (0.85%) | 0.0006 |
| Valproate | 14693 (2.09%) | 236 (6.72%) | 0.2270 |  | 237 (6.74%) | 236 (6.72%) | 0.0007 |
| Cilostazol | 20851 (2.97%) | 106 (3.02%) | 0.0029 |  | 107 (3.03%) | 106 (3.02%) | 0.0008 |
| Dipyridamole | 46129 (6.57%) | 228 (6.49%) | 0.0031 |  | 228 (6.5%) | 228 (6.49%) | 0.0003 |
| Pentoxifylline | 53823 (7.67%) | 376 (10.71%) | 0.1055 |  | 376 (10.71%) | 376 (10.71%) | 0.0002 |

ASMD: Absolute standardized mean difference.

**Supplementary Table 10*.*** Major bleeding risk among patients with AF taking direct oral anticoagulants (DOACs) with or without concurrent antidepressants.

| **Concurrent medication** | **Person-Quarters with DOAC use** | **No. of Bleeding Events** | **Crude Major Bleeding Incidence Rate (95% CI) per 1000 Person-Years** | | ***Adjusted Incidence Rate (95% CI) per 1000 Person-Years** | | ***Adjusted Rate Ratio (95% CI)** | |
| --- | --- | --- | --- | --- | --- | --- | --- | --- |
| Agomelatine |  |  |  |  |  |  |  |  |
| with | 1693 | 13 | 30.61 | (16.79-55.79) | 31.84 | (17.78-57.02) | 0.63 | (0.35-1.13) |
| †without | 703 828 | 8024 | 46.09 | (44.99-47.21) | 50.48 | (48.79-52.22) | 1 |  |
| Amitriptyline |  |  |  |  |  |  |  |  |
| with | 2329 | 37 | 63.55 | (45.73-88.32) | 64.23 | (46.25-89.21) | 1.25 | (0.90-1.74) |
| †without | 703 192 | 8000 | 45.99 | (44.89-47.12) | 51.32 | (49.01-53.73) | 1 |  |
| Bupropion |  |  |  |  |  |  |  |  |
| With | 1457 | 26 | 71.61 | (49.25-104.11) | 71.8 | (49.55-104.04) | #1.49 | (1.02-2.16) |
| †without | 704 064 | 8011 | 46 | (44.90-47.12) | 48.33 | (46.75-49.97) | 1 |  |
| Citalopram |  |  |  |  |  |  |  |  |
| with | 11 838 | 159 | 53.94 | (45.91-63.38) | 54.44 | (46.42-63.85) | 1.07 | (0.91-1.26) |
| †without | 693 683 | 7878 | 45.91 | (44.81-47.05) | 50.72 | (49.26-52.23) | 1 |  |
| Duloxetine |  |  |  |  |  |  |  |  |
| with | 4694 | 72 | 61.79 | (48.54-78.65) | 62.33 | (49.07-79.16) | 1.18 | (0.92-1.51) |
| †without | 700 827 | 7965 | 45.94 | (44.84-47.07) | 52.75 | (49.57-56.14) | 1 |  |
| Escitalopram |  |  |  |  |  |  |  |  |
| with | 10 825 | 148 | 54.91 | (46.50-64.84) | 55.44 | (47.03-65.36) | 1.09 | (0.92-1.29) |
| †without | 694 696 | 7889 | 45.91 | (44.81-47.04) | 50.91 | (49.42-52.44) | 1 |  |
| Fluoxetine |  |  |  |  |  |  |  |  |
| with | 2653 | 28 | 41.89 | (28.39-61.81) | 42.64 | (29.09-62.50) | 0.83 | (0.57-1.22) |
| †without | 702 868 | 8009 | 46.07 | (44.96-47.19) | 51.10 | (49.49-52.76) | 1 |  |
| Imipramine |  |  |  |  |  |  |  |  |
| with | 15 429 | 194 | 50.31 | (43.48-58.20) | 50.79 | (43.96-58.68) | 0.99 | (0.86-1.15) |
| †without | 690 092 | 7843 | 45.95 | (44.85-47.09) | 51.06 | (49.60-52.56) | 1 |  |
| Melitracen |  |  |  |  |  |  |  |  |
| with | 7804 | 92 | 47.39 | (38.00-59.09) | 47.68 | (38.26-59.41) | 0.98 | (0.78-1.22) |
| †without | 697 717 | 7945 | 46.03 | (44.93-47.16) | 48.85 | (47.32-50.44) | 1 |  |
| Mirtazapine |  |  |  |  |  |  |  |  |
| with | 4819 | 66 | 54.26 | (41.93-70.20) | 55.60 | (43.17-71.62) | 1.06 | (0.82-1.36) |
| †without | 700 702 | 7971 | 45.99 | (44.89-47.12) | 52.68 | (51.15-54.24) | 1 |  |
| Paroxetine |  |  |  |  |  |  |  |  |
| with | 2601 | 39 | 60.15 | (43.46-83.26) | 60.35 | (43.68-83.37) | 1.24 | (0.90-1.71) |
| †without | 702 920 | 7998 | 46.00 | (44.90-47.12) | 48.72 | (47.23-50.26) | 1 |  |
| Sertraline |  |  |  |  |  |  |  |  |
| with | 8572 | 109 | 50.34 | (41.24-61.44) | 51.27 | (42.13-62.39) | 0.96 | (0.79-1.17) |
| †without | 696 949 | 7928 | 46.00 | (44.89-47.13) | 53.38 | (51.69-55.13) | 1 |  |
| Trazodone |  |  |  |  |  |  |  |  |
| with | 14 773 | 208 | 56.46 | (48.91-65.18) | 57.16 | (49.59-65.89) | 1.09 | (0.95-1.26) |
| †without | 690 748 | 7829 | 45.83 | (44.72-46.96) | 52.31 | (50.82-53.84) | 1 |  |
| Venlafaxine |  |  |  |  |  |  |  |  |
| with | 1981 | 25 | 50.84 | (33.75-76.58) | 50.97 | (34.01-76.39) | 1.05 | (0.70-1.58) |
| †without | 703 540 | 8012 | 46.04 | (44.94-47.16) | 48.35 | (46.69-50.08) | 1 |  |

*Adjusted by inverse probability of treatment weighting using the propensity score (gender, age, medical utilization, hypertension, myocardial infarction, congestive heart failure, percutaneous coronary intervention, coronary bypass surgery, peripheral vascular disease, cerebrovascular disease, ischemic stroke, transient ischemic attack, hemiplegia or paraplegia, dementia, epilepsy, diabetes mellitus, chronic kidney disease, chronic pulmonary disease, peptic ulcer disease, liver disease, malignancy, anemia, rheumatic disease, human immunodeficiency virus infection, antibiotics and antifungal drugs, antiepileptics, antihypertensives, antiplatelets, bisphosphate, cardiovascular drugs, cyclosporine, glucocorticoid, insulin, lipid lower drugs, nonsteroid anti-inflammatory drugs, residence, income level, and occupation; see **Table 1** and **Supplementary Table 2-4**).

†“without” indicates DOAC alone.

#*P* < 0.05, compared with DOAC alone.

**Supplementary Table 11.** Risk of intracerebral hemorrhage among patients with AF taking direct oral anticoagulants (DOACs) with or without concurrent antidepressants.

| **Concurrent medication** | **Person- Quarters with DOAC use** | **No. of Bleeding Events** | **Crude Major Bleeding Incidence Rate (95% CI) per 1000 Person-Years** | | ***Adjusted Incidence Rate (95% CI) per 1000 Person-Years** | | ***Adjusted Rate Ratio (95% CI)** | |
| --- | --- | --- | --- | --- | --- | --- | --- | --- |
| Agomelatine |  |  |  |  |  |  |  |  |
| with | 1693 | 0 |  |  |  |  |  |  |
| †without | 703 828 | 1264 |  |  |  |  |  |  |
| Amitriptyline |  |  |  |  |  |  |  |  |
| with | 2329 | 6 | 10.42 | (4.70-23.09) | 10.35 | (4.66-23.00) | 1.40 | (0.63-3.11) |
| ††ithout | 703 192 | 1258 | 7.24 | (6.83-7.67) | 7.41 | (6.85-8.02) | 1 |  |
| Bupropion |  |  |  |  |  |  |  |  |
| with | 1457 | 4 | 11.16 | (4.20-29.65) | 11.09 | (4.17-29.47) | 1.40 | (0.52-3.72) |
| †without | 704 064 | 1260 | 7.24 | (6.83-7.67) | 7.95 | (7.38-8.56) | 1 |  |
| Citalopram |  |  |  |  |  |  |  |  |
| With | 11 838 | 35 | 11.94 | (8.46-16.86) | 12.03 | (8.55-16.91) | 1.40 | (0.98-1.98) |
| †without | 693 683 | 1229 | 7.17 | (6.76-7.60) | 8.62 | (8.00-9.29) | 1 |  |
| Duloxetine |  |  |  |  |  |  |  |  |
| with | 4694 | 9 | 7.88 | (4.12-15.07) | 7.77 | (4.05-14.92) | 1.00 | (0.52-1.92) |
| ††ithout | 700 827 | 1255 | 7.24 | (6.84-7.67) | 7.79 | (7.18-8.46) | 1 |  |
| Escitalopram |  |  |  |  |  |  |  |  |
| With | 10 825 | 30 | 11.17 | (7.77-16.06) | 11.27 | (7.88-16.13) | 1.29 | (0.89-1.86) |
| †without | 694 696 | 1234 | 7.19 | (6.78-7.62) | 8.76 | (8.12-9.46) | 1 |  |
| Fluoxetine |  |  |  |  |  |  |  |  |
| with | 2653 | 6 | 9.01 | (3.97-20.44) | 9.15 | (4.10-20.37) | 1.05 | (0.47-2.36) |
| †without | 702 868 | 1258 | 7.24 | (6.84-7.67 | 8.68 | (8.06-9.35) | 1 |  |
| Imipramine |  |  |  |  |  |  |  |  |
| With | 15 429 | 38 | 9.91 | (7.21-13.62) | 9.91 | (7.22-13.60) | 1.31 | (0.95-1.81) |
| †without | 690 092 | 1226 | 7.19 | (6.78-7.62) | 7.57 | (7.09-8.08) | 1 |  |
| Melitracen |  |  |  |  |  |  |  |  |
| with | 7804 | 11 | 5.74 | (3.19-10.33) | 5.67 | (3.15-10.22) | 0.81 | (0.45-1.46) |
| †without | 697 717 | 1253 | 7.26 | (6.86-7.70) | 7.02 | (6.54-7.52) | 1 |  |
| Mirtazapine |  |  |  |  |  |  |  |  |
| with | 4819 | 14 | 11.67 | (6.91-19.71) | 11.72 | (6.96-19.75) | 1.45 | (0.85-2.45) |
| †without | 700 702 | 1250 | 7.22 | (6.81-7.65) | 8.10 | (7.56-8.69) | 1 |  |
| Paroxetine |  |  |  |  |  |  |  |  |
| With | 2601 | 11 | 16.95 | (9.45-30.39) | 16.9 | (9.41-30.36) | #2.11 | (1.17-3.81) |
| †without | 702 920 | 1253 | 7.21 | (6.81-7.64) | 8 | (7.43-8.61) | 1 |  |
| Sertraline |  |  |  |  |  |  |  |  |
| with | 8572 | 25 | 11.6 | (7.69-17.49) | 11.86 | (7.90-17.79) | 1.26 | (0.84 - 1.91) |
| †without | 696 949 | 1239 | 7.19 | (6.79-7.62) | 9.39 | (8.68-10.16) | 1 |  |
| Trazodone |  |  |  |  |  |  |  |  |
| With | 14 773 | 40 | 10.85 | (7.89-14.94) | 10.95 | (7.98-15.03) | 1.34 | (0.97-1.85) |
| †without | 690 748 | 1224 | 7.17 | (6.76-7.60) | 8.17 | (7.63-8.75) | 1 |  |
| Venlafaxine |  |  |  |  |  |  |  |  |
| with | 1981 | 5 | 10.41 | (4.32-25.07) | 10.29 | (4.27-24.80) | 1.31 | (0.54 - 3.18) |
| †without | 703 540 | 1259 | 7.24 | (6.83-7.67) | 7.83 | (7.19-8.54) | 1 |  |

*Adjusted by inverse probability of treatment weighting using the propensity score (gender, age, medical utilization, hypertension, myocardial infarction, congestive heart failure, percutaneous coronary intervention, coronary bypass surgery, peripheral vascular disease, cerebrovascular disease, ischemic stroke, transient ischemic attack, hemiplegia or paraplegia, dementia, epilepsy, diabetes mellitus, chronic kidney disease, chronic pulmonary disease, peptic ulcer disease, liver disease, malignancy, anemia, rheumatic disease, human immunodeficiency virus infection, antibiotics and antifungal drugs, antiepileptics, antihypertensives, antiplatelets, bisphosphate, cardiovascular drugs, cyclosporine, glucocorticoid, insulin, lipid lower drugs, nonsteroid anti-inflammatory drugs, residence, income level, and occupation; see **Table 1** and **Supplementary Table 2-4**).

†“without” indicates DOAC alone.

#*P* < 0.05, compared with DOAC alone.

**Supplementary Table 12.** Risk of gastrointestinal hemorrhage among patients with AF taking direct oral anticoagulants (DOACs) with or without concurrent antidepressants.

| **Concurrent medication** | **Person- Quarters with DOAC use** | **No. of Bleeding Events** | **Crude Major Bleeding Incidence Rate (95% CI) per 1000 Person-Years** | | ***Adjusted Incidence Rate (95% CI) per 1000 Person-Years** | | ***Adjusted Rate Ratio (95% CI)** | |
| --- | --- | --- | --- | --- | --- | --- | --- | --- |
| Agomelatine |  |  |  |  |  |  |  |  |
| with | 1693 | 13 | 30.99 | (17.12-56.10) | 31.87 | (17.81-57.04) | 0.79 | (0.44-1.41) |
| †without | 703 828 | 6477 | 37.15 | (36.16-38.17) | 40.44 | (38.94-42.00) | 1 |  |
| Amitriptyline |  |  |  |  |  |  |  |  |
| with | 2329 | 30 | 51.45 | (35.43-74.73) | 52.25 | (36.05-75.74) | 1.24 | (0.85-1.80) |
| †without | 703 192 | 6460 | 37.09 | (36.09-38.11) | 42.12 | (39.92-44.43) | 1 |  |
| Bupropion |  |  |  |  |  |  |  |  |
| With | 1457 | 22 | 60.23 | (39.94-90.84) | 60.65 | (40.47-90.89) | #1.57 | (1.04-2.35) |
| without | 704 064 | 6468 | 37.09 | (36.09-38.11) | 38.74 | (37.29-40.25) | 1 |  |
| Citalopram |  |  |  |  |  |  |  |  |
| with | 11 838 | 123 | 41.6 | (34.63-49.97) | 41.97 | (34.99-50.32) | 1.04 | (0.86- 1.25) |
| †without | 693 683 | 6367 | 37.06 | (36.06-38.08) | 40.46 | (39.16-41.80) | 1 |  |
| Duloxetine |  |  |  |  |  |  |  |  |
| with | 4694 | 59 | 50.53 | (38.58-66.19) | 51.07 | (39.10-66.71) | 1.18 | (0.89-1.55) |
| †without | 700 827 | 6431 | 37.04 | (36.05-38.07) | 43.32 | (40.22-46.66) | 1 |  |
| Escitalopram |  |  |  |  |  |  |  |  |
| with | 10 825 | 117 | 43.34 | (35.91-52.30) | 43.69 | (36.26-52.65) | 1.08 | (0.89-1.30) |
| †without | 694 696 | 6373 | 37.04 | (36.04-38.06) | 40.49 | (39.17-41.85) | 1 |  |
| Fluoxetine |  |  |  |  |  |  |  |  |
| with | 2653 | 22 | 32.82 | (21.09-51.09) | 33.37 | (21.59-51.58) | 0.82 | (0.53-1.27) |
| †without | 702 868 | 6468 | 37.15 | (36.16-38.17) | 40.73 | (39.28-42.23) | 1 |  |
| Imipramine |  |  |  |  |  |  |  |  |
| with | 15 429 | 154 | 39.8 | (33.73-46.96) | 40.29 | (34.21-47.45) | 0.96 | (0.81-1.14) |
| †without | 690 092 | 6336 | 37.07 | (36.07-38.10) | 41.86 | (40.51-43.25) | 1 |  |
| Melitracen |  |  |  |  |  |  |  |  |
| with | 7804 | 79 | 40.46 | (31.73-51.60) | 40.88 | (32.11-52.05) | 1.02 | (0.80-1.30) |
| †without | 697 717 | 6411 | 37.10 | (36.10-38.12) | 40.19 | (38.75-41.67) | 1 |  |
| Mirtazapine |  |  |  |  |  |  |  |  |
| with | 4819 | 52 | 42.62 | (31.93-56.88) | 43.69 | (32.95-57.95) | 1.02 | (0.77-1.35) |
| †without | 700 702 | 6438 | 37.1 | (36.10-38.12) | 42.86 | (41.47-44.30) | 1 |  |
| Paroxetine |  |  |  |  |  |  |  |  |
| with | 2601 | 28 | 43.19 | (29.45-63.35) | 43.55 | (29.81-63.61) | 1.12 | (0.76-1.63) |
| †without | 702 920 | 6462 | 37.11 | (36.12-38.13) | 39.02 | (37.67-40.42) | 1 |  |
| Sertraline |  |  |  |  |  |  |  |  |
| with | 8572 | 79 | 36.39 | (28.66-46.21) | 37.09 | (29.32-46.91) | 0.87 | (0.69-1.11) |
| †without | 696 949 | 6411 | 37.14 | (36.14-38.17) | 42.40 | (40.90-43.96) | 1 |  |
| Trazodone |  |  |  |  |  |  |  |  |
| with | 14 773 | 159 | 43.18 | (36.73-50.77) | 43.64 | (37.17-51.23) | 1.03 | (0.87-1.21) |
| †without | 690 748 | 6331 | 37.00 | (36.00-38.03) | 42.53 | (41.16-43.93) | 1 |  |
| Venlafaxine |  |  |  |  |  |  |  |  |
| with | 1981 | 20 | 40.15 | (25.27-63.79) | 40.62 | (25.77-64.00) | 1.05 | (0.66-1.65) |
| †without | 703 540 | 6470 | 37.13 | (36.13-38.15) | 38.79 | (37.34-40.31) | 1 |  |

*Adjusted by inverse probability of treatment weighting using the propensity score (gender, age, medical utilization, hypertension, myocardial infarction, congestive heart failure, percutaneous coronary intervention, coronary bypass surgery, peripheral vascular disease, cerebrovascular disease, ischemic stroke, transient ischemic attack, hemiplegia or paraplegia, dementia, epilepsy, diabetes mellitus, chronic kidney disease, chronic pulmonary disease, peptic ulcer disease, liver disease, malignancy, anemia, rheumatic disease, human immunodeficiency virus infection, antibiotics and antifungal drugs, antiepileptics, antihypertensives, antiplatelets, bisphosphate, cardiovascular drugs, cyclosporine, glucocorticoid, insulin, lipid lower drugs, nonsteroid anti-inflammatory drugs, residence, income level, and occupation; see **Table 1**and **Supplementary Table 2-4**).

†“without” indicates DOAC alone.

#*P* < 0.05, compared with DOAC alone.

**Supplementary Table 13.** Risk of intraspinal, intraocular, retroperitoneal, intra-articular, pericardial, or intramuscular hemorrhage patients with AF taking direct oral anticoagulants (DOACs) with or without concurrent antidepressants.

| **Concurrent medication** | **Person-Quarters with DOAC use** | **No. of Bleeding Events** | **Crude Major Bleeding Incidence Rate (95% CI) per 1000 Person-Years** | | ***Adjusted Incidence Rate (95% CI) per 1000 Person-Years** | | ***Adjusted Rate Ratio (95% CI)** | |
| --- | --- | --- | --- | --- | --- | --- | --- | --- |
| SNRI |  |  |  |  |  |  |  |  |
| with | 6642 | 4 | 2.41 | (0.90-6.42) | 2.41 | (0.90-6.42) | 1.50 | (0.56-4.06) |
| †without | 698 879 | 279 | 1.6 | (1.42-1.80) | 1.6 | (1.37-1.88) | 1 |  |
| SSRI |  |  |  |  |  |  |  |  |
| with | 25 344 | 6 | 0.95 | (0.43-2.11) | 0.95 | (0.43-2.11) | 0.58 | (0.26-1.30) |
| †without | 680 177 | 277 | 1.63 | (1.44-1.84) | 1.64 | (1.42-1.89) | 1 |  |
| TCA |  |  |  |  |  |  |  |  |
| with | 25 083 | 5 | 0.80 | (0.33-1.91) | 0.80 | (0.33-1.91) | 0.51 | (0.21-1.23) |
| †without | 680 438 | 278 | 1.63 | (1.45-1.84) | 1.57 | (1.38-1.79) | 1 |  |
| TeCA |  |  |  |  |  |  |  |  |
| with | 19 179 | 9 | 1.88 | (0.98-3.60) | 1.88 | (0.98-3.60) | 1.21 | (0.62-2.35) |
| †without | 686 342 | 274 | 1.6 | (1.41-1.80) | 1.56 | (1.36-1.78) | 1 |  |
| Others |  |  |  |  |  |  |  |  |
| with | 3511 | 0 |  |  |  |  |  |  |
| †without | 702 010 | 283 |  |  |  |  |  |  |
| Agomelatine |  |  |  |  |  |  |  |  |
| with | 1693 | 0 |  |  |  |  |  |  |
| †without | 703 828 | 283 |  |  |  |  |  |  |
| Amitriptyline |  |  |  |  |  |  |  |  |
| with | 2329 | 1 |  |  |  |  |  |  |
| †without | 703 192 | 282 |  |  |  |  |  |  |
| Bupropion |  |  |  |  |  |  |  |  |
| with | 1457 | 0 |  |  |  |  |  |  |
| †without | 704 064 | 283 |  |  |  |  |  |  |
| Citalopram |  |  |  |  |  |  |  |  |
| with | 11 838 | 1 |  |  |  |  |  |  |
| †without | 693 683 | 282 |  |  |  |  |  |  |
| Duloxetine |  |  |  |  |  |  |  |  |
| with | 4694 | 4 | 3.41 | (1.28-9.09) | 3.41 | (1.28-9.09) | 2.17 | (0.80-5.87) |
| †without | 700 827 | 279 | 1.59 | (1.41-1.80) | 1.57 | (1.33-1.85) | 1 |  |
| Escitalopram |  |  |  |  |  |  |  |  |
| with | 10 825 | 1 |  |  |  |  |  |  |
| †without | 694 696 | 282 |  |  |  |  |  |  |
| Fluoxetine |  |  |  |  |  |  |  |  |
| with | 2653 | 0 |  |  |  |  |  |  |
| †without | 702 868 | 283 |  |  |  |  |  |  |
| Imipramine |  |  |  |  |  |  |  |  |
| with | 15 429 | 2 |  |  |  |  |  |  |
| †without | 690 092 | 281 |  |  |  |  |  |  |
| Melitracen |  |  |  |  |  |  |  |  |
| with | 7804 | 2 |  |  |  |  |  |  |
| †without | 697 717 | 281 |  |  |  |  |  |  |
| Mirtazapine |  |  |  |  |  |  |  |  |
| with | 4819 | 0 |  |  |  |  |  |  |
| †without | 700 702 | 283 |  |  |  |  |  |  |
| Paroxetine |  |  |  |  |  |  |  |  |
| with | 2601 | 0 |  |  |  |  |  |  |
| †without | 702 920 | 283 |  |  |  |  |  |  |
| Sertraline |  |  |  |  |  |  |  |  |
| with | 8572 | 5 | 2.33 | (0.97-5.60) | 2.33 | (0.97-5.60) | 1.47 | (0.61-3.59) |
| †without | 696 949 | 278 | 1.60 | (1.41-1.80) | 1.58 | (1.35-1.85) | 1 |  |
| Trazodone |  |  |  |  |  |  |  |  |
| with | 14 773 | 9 | 2.44 | (1.27-4.68) | 2.44 | (1.27-4.68) | 1.58 | (0.81-3.07) |
| †without | 690 748 | 274 | 1.59 | (1.41-1.79) | 1.55 | (1.35-1.77) | 1 |  |
| Venlafaxine |  |  |  |  |  |  |  |  |
| with | 1981 | 0 |  |  |  |  |  |  |
| †without | 703 540 | 283 |  |  |  |  |  |  |

*Adjusted by inverse probability of treatment weighting using the propensity score (gender, age, medical utilization, hypertension, myocardial infarction, congestive heart failure, percutaneous coronary intervention, coronary bypass surgery, peripheral vascular disease, cerebrovascular disease, ischemic stroke, transient ischemic attack, hemiplegia or paraplegia, dementia, epilepsy, diabetes mellitus, chronic kidney disease, chronic pulmonary disease, peptic ulcer disease, liver disease, malignancy, anemia, rheumatic disease, human immunodeficiency virus infection, antibiotics and antifungal drugs, antiepileptics, antihypertensives, antiplatelets, bisphosphate, cardiovascular drugs, cyclosporine, glucocorticoid, insulin, lipid lower drugs, nonsteroid anti-inflammatory drugs, residence, income level, and occupation; see **Table 1** and **Supplementary Table 2-4**).

†without indicates DOAC alone.

**Supplementary Table 14.** Bleeding risk among patients with AF taking dabigatran with or without concurrent antidepressants.

| **Concurrent medication** | **Person-Quarters with DOAC use** | **No. of Bleeding Events** | **Crude Major Bleeding Incidence Rate (95% CI) per 1000 Person-Years** | | ***Adjusted Incidence Rate (95% CI) per 1000 Person-Years** | | ***Adjusted Rate Ratio (95% CI)** | |
| --- | --- | --- | --- | --- | --- | --- | --- | --- |
| **Major bleeding** | |  |  |  |  |  |  |  |
| SNRI |  |  |  |  |  |  |  |  |
| with | 2665 | 39 | 59.57 | (43.42-81.72) | 59.13 | (43.14-81.04) | 1.32 | (0.96-1.83) |
| †without | 267 899 | 2678 | 40.49 | (38.87-42.17) | 44.64 | (41.81-47.66) | 1 |  |
| SSRI |  |  |  |  |  |  |  |  |
| with | 9680 | 119 | 49.27 | (40.70-59.65) | 49.77 | (41.17-60.17) | 1.09 | (0.90-1.32) |
| †without | 260 884 | 2598 | 40.35 | (38.72-42.05) | 45.77 | (43.58-48.08) | 1 |  |
| TCA |  |  |  |  |  |  |  |  |
| with | 10 242 | 116 | 45.58 | (37.80-54.96) | 45.65 | (37.87-55.03) | 1.05 | (0.86-1.27) |
| †without | 260 322 | 2601 | 40.48 | (38.84-42.18) | 43.61 | (41.64-45.68) | 1 |  |
| TeCA |  |  |  |  |  |  |  |  |
| with | 7252 | 98 | 54.2 | (43.76-67.12) | 54.62 | (44.16-67.57) | 1.19 | (0.95-1.47) |
| †without | 263 312 | 2619 | 40.3 | (38.68-41.98) | 46.09 | (43.90-48.39) | 1 |  |
| Others |  |  |  |  |  |  |  |  |
| with | 1293 | 12 | 36.75 | (20.00- 67.54) | 37.44 | (20.56- 68.19) | 0.9 | (0.49 - 1.65) |
| †without | 269 271 | 2705 | 40.69 | (39.08- 42.38) | 41.48 | (39.31- 43.78) | 1 |  |
| **Intracerebral hemorrhage** | |  |  |  |  |  |  |  |
| SNRI |  |  |  |  |  |  |  |  |
| with | 2665 | 7 | 10.71 | (5.12-22.39) | 10.59 | (5.05-22.21) | 1.55 | (0.72-3.35) |
| †without | 267 899 | 383 | 5.78 | (5.21-6.40) | 6.81 | (5.59-8.31) | 1 |  |
| SSRI |  |  |  |  |  |  |  |  |
| with | 9680 | 26 | 10.78 | (7.31-15.90) | 10.9 | (7.41-16.03) | #1.55 | (1.04-2.33) |
| †without | 260 884 | 364 | 5.64 | (5.07-6.26) | 7.01 | (6.15-8.00) | 1 |  |
| TCA |  |  |  |  |  |  |  |  |
| with | 10 242 | 22 | 8.63 | (5.68-13.11) | 8.64 | (5.70-13.11) | 1.46 | (0.95-2.25) |
| †without | 260 322 | 368 | 5.71 | (5.14-6.34) | 5.91 | (5.27-6.63) | 1 |  |
| TeCA |  |  |  |  |  |  |  |  |
| with | 7252 | 15 | 8.29 | (4.85-14.19) | 8.32 | (4.87-14.22) | 1.27 | (0.73-2.19) |
| †without | 263 312 | 375 | 5.75 | (5.19-6.38) | 6.56 | (5.81-7.40) | 1 |  |
| Others |  |  |  |  |  |  |  |  |
| with | 1293 | 0 |  |  |  |  |  |  |
| †without | 269 271 | 390 |  |  |  |  |  |  |
| **Gastrointestinal hemorrhage** | |  |  |  |  |  |  |  |
| SNRI |  |  |  |  |  |  |  |  |
| with | 2665 | 29 | 44.22 | (30.65-63.79) | 43.98 | (30.53-63.35) | 1.21 | (0.83-1.75) |
| †without | 267 899 | 2202 | 33.27 | (31.80-34.80) | 36.47 | (34.00-39.12) | 1 |  |
| SSRI |  |  |  |  |  |  |  |  |
| with | 9680 | 91 | 37.58 | (30.17-46.81) | 37.97 | (30.55-47.21) | 1.01 | (0.81-1.27) |
| †without | 260 884 | 2140 | 33.22 | (31.74-34.77) | 37.43 | (35.45-39.52) | 1 |  |
| TCA |  |  |  |  |  |  |  |  |
| with | 10 242 | 92 | 36.08 | (29.16-44.65) | 36.16 | (29.23-44.72) | 1 | (0.80-1.24) |
| †without | 260 322 | 2139 | 33.27 | (31.78-34.82) | 36.24 | (34.42-38.16) | 1 |  |
| TeCA |  |  |  |  |  |  |  |  |
| with | 7252 | 79 | 43.68 | (34.63-55.10) | 43.98 | (34.92-55.40) | 1.15 | (0.91-1.46) |
| †without | 263 312 | 2152 | 33.09 | (31.62-34.63) | 38.16 | (36.14-40.29) | 1 |  |
| Others |  |  |  |  |  |  |  |  |
| with | 1293 | 12 | 36.93 | (20.16-67.64) | 37.47 | (20.59-68.21) | 1.09 | (0.60-1.99) |
| †without | 269 271 | 2219 | 33.36 | (31.89-34.89) | 34.32 | (32.32-36.45) | 1 |  |
| **Intraspinal, intraocular, retroperitoneal, intra-articular, pericardial, or intramuscular hemorrhage** | | | | | | | | |
| SNRI |  |  |  |  |  |  |  |  |
| with | 2665 | 3 |  |  |  |  |  |  |
| †without | 267 899 | 93 |  |  |  |  |  |  |
| SSRI |  |  |  |  |  |  |  |  |
| with | 9680 | 2 |  |  |  |  |  |  |
| †without | 260884 | 94 |  |  |  |  |  |  |
| TCA |  |  |  |  |  |  |  |  |
| with | 10 242 | 2 |  |  |  |  |  |  |
| †without | 260 322 | 94 |  |  |  |  |  |  |
| TeCA |  |  |  |  |  |  |  |  |
| with | 7252 | 4 | 2.21 | (0.83-5.87) | 2.21 | (0.83-5.87) | 1.75 | (0.64-4.80) |
| †without | 263 312 | 92 | 1.40 | (1.13-1.72) | 1.26 | (0.99-1.59) | 1 |  |
| Others |  |  |  |  |  |  |  |  |
| with | 1293 | 0 |  |  |  |  |  |  |
| †without | 269 271 | 96 |  |  |  |  |  |  |

*Adjusted by inverse probability of treatment weighting using the propensity score (gender, age, medical utilization, hypertension, myocardial infarction, congestive heart failure, percutaneous coronary intervention, coronary bypass surgery, peripheral vascular disease, cerebrovascular disease, ischemic stroke, transient ischemic attack, hemiplegia or paraplegia, dementia, epilepsy, diabetes mellitus, chronic kidney disease, chronic pulmonary disease, peptic ulcer disease, liver disease, malignancy, anemia, rheumatic disease, human immunodeficiency virus infection, antibiotics and antifungal drugs, antiepileptics, antihypertensives, antiplatelets, bisphosphate, cardiovascular drugs, cyclosporine, glucocorticoid, insulin, lipid lower drugs, nonsteroid anti-inflammatory drugs, residence, income level, and occupation; see **Table 1** and **Supplementary Table 2-4**).

†without indicates dabigatran alone.

**Supplementary Table 15.** Bleeding risk among patients with AF taking apixaban with or without concurrent antidepressants.

| **Concurrent medication** | **Person-Quarters with DOAC use** | **No. of Bleeding Events** | **Crude Major Bleeding Incidence Rate (95% CI) per 1000 Person-Years** | | ***Adjusted Incidence Rate (95% CI) per 1000 Person-Years** | | ***Adjusted Rate Ratio (95% CI)** | |
| --- | --- | --- | --- | --- | --- | --- | --- | --- |
| **Major bleeding** | |  |  |  |  |  |  |  |
| SNRI |  |  |  |  |  |  |  |  |
| with | 813 | 19 | 95.03 | (61.46-146.94) | 94.32 | (60.87-146.17) | #1.63 | (1.04-2.55) |
| †without | 89 336 | 1079 | 49.05 | (46.00-52.30) | 57.76 | (50.47-66.11) | 1 |  |
| SSRI |  |  |  |  |  |  |  |  |
| with | 3385 | 44 | 52.39 | (38.97-70.42) | 52.48 | (39.14-70.36) | 0.95 | (0.70-1.28) |
| †without | 86 764 | 1054 | 49.34 | (46.24-52.65) | 55.52 | (51.12-60.29) | 1 |  |
| TCA |  |  |  |  |  |  |  |  |
| with | 3127 | 43 | 55.22 | (40.63-75.05) | 55.57 | (41.01-75.30) | 1.1 | (0.80-1.50) |
| †without | 87 022 | 1055 | 49.25 | (46.15-52.55) | 50.74 | (47.19-54.55) | 1 |  |
| TeCA |  |  |  |  |  |  |  |  |
| with | 2477 | 39 | 63.62 | (45.97-88.03) | 63.74 | (46.04-88.23) | 1.12 | (0.80-1.57) |
| †without | 87 672 | 1059 | 49.06 | (45.98-52.33) | 56.75 | (52.43-61.43) | 1 |  |
| Others |  |  |  |  |  |  |  |  |
| with | 511 | 6 | 46.19 | (20.27-105.24) | 47.32 | (21.32-105.02) | 0.86 | (0.39-1.92) |
| †without | 89 638 | 1092 | 49.47 | (46.41-52.73) | 54.93 | (50.03-60.31) | 1 |  |
| **Intracerebral hemorrhage** | |  |  |  |  |  |  |  |
| SNRI |  |  |  |  |  |  |  |  |
| with | 813 | 20 | 10.31 | (2.61-40.70) | 10.11 | (2.52-40.50) | 1.05 | (0.26-4.26) |
| †without | 89 336 | 183 | 9.13 | (7.90-10.55) | 9.67 | (7.95-11.76) | 1 |  |
| SSRI |  |  |  |  |  |  |  |  |
| with | 3385 | 13 | 15.51 | (8.96-26.84) | 15.64 | (9.08-26.94) | 1.40 | (0.78-2.51) |
| †without | 86 764 | 190 | 8.89 | (7.66-10.32) | 11.15 | (9.07-13.70) | 1 |  |
| TCA |  |  |  |  |  |  |  |  |
| with | 3127 | 10 | 12.92 | (6.91-24.17) | 12.89 | (6.93-23.97) | 1.47 | (0.77-2.79) |
| †without | 87 022 | 193 | 9.00 | (7.77-10.44) | 8.78 | (7.46-10.34) | 1 |  |
| TeCA |  |  |  |  |  |  |  |  |
| with | 2477 | 10 | 16.58 | (8.96-30.69) | 16.46 | (8.86-30.60) | 1.62 | (0.85-3.09) |
| †without | 87 672 | 193 | 8.93 | (7.71-10.35) | 10.17 | (8.47-12.21) | 1 |  |
| Others |  |  |  |  |  |  |  |  |
| with | 511 | 2 |  |  |  |  |  |  |
| †without | 89 638 | 201 |  |  |  |  |  |  |
| **Gastrointestinal hemorrhage** | |  |  |  |  |  |  |  |
| SNRI |  |  |  |  |  |  |  |  |
| with | 813 | 15 | 74.72 | (45.73-122.08) | 74.33 | (45.42-121.65) | 1.59 | (0.96-2.63) |
| †without | 89 336 | 848 | 38.52 | (35.80-41.44) | 46.65 | (39.72-54.79) | 1 |  |
| SSRI |  |  |  |  |  |  |  |  |
| with | 3385 | 31 | 36.74 | (25.80-52.31) | 36.88 | (26.00-52.32) | 0.86 | (0.60-1.24) |
| †without | 86 764 | 832 | 38.92 | (36.16-41.90) | 42.72 | (38.96-46.85) | 1 |  |
| TCA |  |  |  |  |  |  |  |  |
| with | 3127 | 32 | 40.88 | (28.56-58.52) | 41.25 | (28.92-58.83) | 1.01 | (0.71-1.46) |
| †without | 87 022 | 831 | 38.76 | (36.01-41.73) | 40.66 | (37.45-44.14) | 1 |  |
| TeCA |  |  |  |  |  |  |  |  |
| with | 2477 | 28 | 45.24 | (30.58-66.94) | 45.64 | (30.89-67.45) | 1.01 | (0.68-1.51) |
| †without | 87 672 | 835 | 38.66 | (35.92-41.60) | 45.04 | (41.16-49.29) | 1 |  |
| Others |  |  |  |  |  |  |  |  |
| with | 511 | 4 | 30.67 | (11.18-84.17) | 31.41 | (11.79-83.70) | 0.75 | (0.28-2.00) |
| †without | 89 638 | 859 | 38.89 | (36.16-41.81) | 42.07 | (37.91-46.68) | 1 |  |
| **Intraspinal, intraocular, retroperitoneal, intra-articular, pericardial, or intramuscular hemorrhage** | | | | | | | | |
| SNRI |  |  |  |  |  |  |  |  |
| with | 813 | 2 |  |  |  |  |  |  |
| †without | 89 336 | 30 |  |  |  |  |  |  |
| SSRI |  |  |  |  |  |  |  |  |
| with | 3385 | 0 |  |  |  |  |  |  |
| †without | 86 764 | 32 |  |  |  |  |  |  |
| TCA |  |  |  |  |  |  |  |  |
| with | 3127 | 1 |  |  |  |  |  |  |
| †without | 87 022 | 31 |  |  |  |  |  |  |
| TeCA |  |  |  |  |  |  |  |  |
| with | 2477 | 1 |  |  |  |  |  |  |
| †without | 87 672 | 31 |  |  |  |  |  |  |
| Others |  |  |  |  |  |  |  |  |
| with | 511 | 0 |  |  |  |  |  |  |
| †without | 89 638 | 32 |  |  |  |  |  |  |

*Adjusted by inverse probability of treatment weighting using the propensity score (gender, age, medical utilization, hypertension, myocardial infarction, congestive heart failure, percutaneous coronary intervention, coronary bypass surgery, peripheral vascular disease, cerebrovascular disease, ischemic stroke, transient ischemic attack, hemiplegia or paraplegia, dementia, epilepsy, diabetes mellitus, chronic kidney disease, chronic pulmonary disease, peptic ulcer disease, liver disease, malignancy, anemia, rheumatic disease, human immunodeficiency virus infection, antibiotics and antifungal drugs, antiepileptics, antihypertensives, antiplatelets, bisphosphate, cardiovascular drugs, cyclosporine, glucocorticoid, insulin, lipid lower drugs, nonsteroid anti-inflammatory drugs, residence, income level, and occupation; see **Table 1** and **Supplementary Table 2-4**).

†without indicates apixaban alone.

**Supplementary Table 16.** Bleeding risk among patients with AF taking rivaroxaban with or without concurrent antidepressants.

| **Concurrent medication** | **Person-Quarters with DOAC use** | **No. of Bleeding Events** | **Crude Major Bleeding Incidence Rate (95% CI) per 1000 Person-Years** | | ***Adjusted Incidence Rate (95% CI) per 1000 Person-Years** | | ***Adjusted Rate Ratio (95% CI)** | | |
| --- | --- | --- | --- | --- | --- | --- | --- | --- | --- |
| **Major bleeding** | |  |  |  |  |  |  |  | |
| SNRI |  |  |  |  |  |  |  |  | |
| with | 3200 | 37 | 46.20 | (33.01-64.66) | 46.72 | (33.48-65.18) | 0.83 | (0.59-1.17) | |
| †without | 334 492 | 4141 | 50.16 | (48.53-51.85) | 56.00 | (52.56-59.66) | 1 |  | |
| SSRI |  |  |  |  |  |  |  |  | |
| with | 12 421 | 159 | 51.22 | (43.57-60.21) | 51.56 | (43.92-60.53) | 0.91 | (0.78-1.08) | |
| †without | 325 271 | 4019 | 50.08 | (48.42-51.80) | 56.39 | (54.08-58.80) | 1 |  | |
| TCA |  |  |  |  |  |  |  |  | |
| with | 11 653 | 157 | 54.20 | (45.92-63.97) | 54.46 | (46.17-64.25) | 0.98 | (0.83-1.16) | |
| †without | 326 039 | 4021 | 49.98 | (48.33-51.69) | 55.49 | (53.32-57.75) | 1 |  | |
| TeCA |  |  |  |  |  |  |  |  | |
| with | 9506 | 133 | 56.51 | (47.51-67.22) | 56.81 | (47.82-67.48) | 0.99 | (0.83-1.19) | |
| †without | 328 186 | 4045 | 49.94 | (48.29-51.64) | 57.10 | (54.91-59.37) | 1 |  | |
| Others |  |  |  |  |  |  |  |  | |
| with | 1727 | 23 | 54.67 | (36.49-81.92) | 54.02 | (36.02-81.02) | 1.01 | (0.67-1.51) | |
| †without | 335 965 | 4155 | 50.10 | (48.47-51.79) | 53.67 | (51.39-56.06) | 1 |  | |
| **Intracerebral hemorrhage** | |  |  |  |  |  |  |  | |
| SNRI |  |  |  |  |  |  |  |  | |
| with | 3200 | 4 | 5.11 | (1.93-13.55) | 5.05 | (1.89-13.46) | 0.58 | (0.22-1.57) | |
| †without | 334 492 | 686 | 8.30 | (7.68-8.97) | 8.64 | (7.80-9.58) | 1 |  | |
| SSRI |  |  |  |  |  |  |  |  | |
| with | 12 421 | 35 | 11.25 | (7.92-15.98) | 11.37 | (8.03-16.10) | 1.12 | (0.78 -1.61) | |
| †without | 325 271 | 655 | 8.16 | (7.53-8.83) | 10.14 | (9.15-11.22) | 1 |  | |
| TCA |  |  |  |  |  |  |  |  | |
| with | 11 653 | 23 | 7.98 | (5.32-11.99) | 7.93 | (5.28-11.92) | 0.94 | (0.62-1.43) | |
| †without | 326 039 | 667 | 8.28 | (7.65-8.96) | 8.44 | (7.73-9.21) | 1 |  | |
| TeCA |  |  |  |  |  |  |  |  | |
| with | 9506 | 29 | 12.25 | (8.50-17.64) | 12.28 | (8.55-17.65) | 1.36 | (0.94-1.98) | |
| †without | 328 186 | 661 | 8.16 | (7.54-8.83) | 9.02 | (8.23-9.89) | 1 |  | |
| Others |  |  |  |  |  |  |  |  | |
| with | 1727 | 3 | 7.03 | (2.26-21.91) | 7.05 | (2.27-21.83) | 0.74 | (0.24-2.29) | |
| †without | 335 965 | 687 | 8.28 | (7.66-8.95) | 9.57 | (8.58-10.68) | 1 |  | |
| **Gastrointestinal hemorrhage** | |  |  |  |  |  |  |  | |
| SNRI |  |  |  |  |  |  |  |  | |
| with | 3200 | 33 | 41.02 | (28.64-58.74) | 41.60 | (29.19-59.30) | 0.92 | (0.64-1.32) | |
| †without | 334 492 | 3290 | 39.81 | (38.35-41.33) | 45.12 | (41.84-48.65) | 1 |  | |
| SSRI |  |  |  |  |  |  |  |  | |
| with | 12 421 | 119 | 38.38 | (31.81-46.32) | 38.58 | (32.01-46.49) | 0.87 | (0.72-1.06) | |
| †without | 325 271 | 3204 | 39.88 | (38.39-41.42) | 44.16 | (42.14-46.29) | 1 |  | |
| TCA |  |  |  |  |  |  |  |  | |
| with | 11 653 | 131 | 45.14 | (37.55-54.26) | 45.45 | (37.85-54.58) | 1.01 | (0.84-1.22) | |
| †without | 326 039 | 3192 | 39.63 | (38.15-41.17) | 45.03 | (43.02-47.13) | 1 |  | |
| TeCA |  |  |  |  |  |  |  |  | |
| with | 9506 | 99 | 42.08 | (34.43-51.42) | 42.27 | (34.65-51.56) | 0.92 | (0.75-1.12) | |
| †without | 328 186 | 3224 | 39.76 | (38.28-41.30) | 46.06 | (44.07-48.15) | 1 |  | |
| Others |  |  |  |  |  |  |  |  | |
| with | 1727 | 20 | 47.62 | (30.84-73.55) | 46.88 | (30.34-72.45) | 1.12 | (0.72-1.73) | |
| †without | 335 965 | 3303 | 39.78 | (38.32-41.30) | 42.00 | (40.01-44.08) | 1 |  | |
| **Intraspinal, intraocular, retroperitoneal, intra-articular, pericardial, or intramuscular hemorrhage** | | | | | | | | |  |
| SNRI |  |  |  |  |  |  |  |  | |
| with | 3200 | 0 |  |  |  |  |  |  | |
| †without | 334 492 | 165 |  |  |  |  |  |  | |
| SSRI |  |  |  |  |  |  |  |  | |
| with | 12 421 | 5 | 1.61 | (0.67-3.87) | 1.61 | (0.67-3.87) | 0.78 | (0.32-1.92) | |
| †without | 325 271 | 160 | 1.97 | (1.68-2.31) | 2.06 | (1.70-2.51) | 1 |  | |
| TCA |  |  |  |  |  |  |  |  | |
| with | 11 653 | 3 |  |  |  |  |  |  | |
| †without | 326 039 | 162 |  |  |  |  |  |  | |
| TeCA |  |  |  |  |  |  |  |  | |
| with | 9506 | 5 | 2.10 | (0.88-5.05) | 2.10 | (0.88-5.05) | 1.09 | (0.45-2.67) | |
| †without | 328 186 | 160 | 1.95 | (1.66-2.28) | 1.93 | (1.61-2.31) | 1 |  | |
| Others |  |  |  |  |  |  |  |  | |
| with | 1727 | 0 |  |  |  |  |  |  | |
| †without | 335 965 | 165 |  |  |  |  |  |  | |

*Adjusted by inverse probability of treatment weighting using the propensity score (gender, age, medical utilization, hypertension, myocardial infarction, congestive heart failure, percutaneous coronary intervention, coronary bypass surgery, peripheral vascular disease, cerebrovascular disease, ischemic stroke, transient ischemic attack, hemiplegia or paraplegia, dementia, epilepsy, diabetes mellitus, chronic kidney disease, chronic pulmonary disease, peptic ulcer disease, liver disease, malignancy, anemia, rheumatic disease, human immunodeficiency virus infection, antibiotics and antifungal drugs, antiepileptics, antihypertensives, antiplatelets, bisphosphate, cardiovascular drugs, cyclosporine, glucocorticoid, insulin, lipid lower drugs, nonsteroid anti-inflammatory drugs, residence, income level, and occupation; see **Table 1** and **Supplementary Table 2-4**).

†without indicates rivaroxaban alone.

**Supplementary Table 17.** Risk of acute pancreatitis and acute appendicitis among patients with AF taking direct oral anticoagulants (DOACs) with or without concurrent antidepressants.

| **Concurrent medication** | **Person-Quarters with DOAC use** | **No. of Events** | **Crude Event Incidence Rate (95% CI) per 1000 Person-Years** | | ***Adjusted Incidence Rate (95% CI) per 1000 Person-Years** | | ***Adjusted Rate Ratio (95% CI)** | |
| --- | --- | --- | --- | --- | --- | --- | --- | --- |
| **Acute pancreatitis** | |  |  |  |  |  |  |  |
| SNRI |  |  |  |  |  |  |  |  |
| with | 6658 | 1 |  |  |  |  |  |  |
| †without | 698 863 | 223 |  |  |  |  |  |  |
| SSRI |  |  |  |  |  |  |  |  |
| with | 25 405 | 11 | 1.73 | (0.91-3.29) | 1.74 | (0.92-3.31) | 1.24 | (0.65-2.38) |
| †without | 680 116 | 213 | 1.25 | (1.08-1.46) | 1.41 | (1.17-1.68) | 1 |  |
| TCA |  |  |  |  |  |  |  |  |
| with | 25 137 | 11 | 1.76 | (0.96-3.20) | 1.77 | (0.98-3.19) | 1.29 | (0.70-2.38) |
| †without | 680 384 | 213 | 1.25 | (1.08-1.46) | 1.37 | (1.16-1.62) | 1 |  |
| TeCA |  |  |  |  |  |  |  |  |
| with | 19 223 | 8 | 1.60 | (0.52-4.96) | 1.63 | (0.53-5.04) | 1.10 | (0.36-3.36) |
| †without | 686 298 | 216 | 1.26 | (1.09-1.46) | 1.49 | (1.26-1.75) | 1 |  |
| Others |  |  |  |  |  |  |  |  |
| with | 3522 | 3 |  |  |  |  |  |  |
| †without | 701 999 | 221 |  |  |  |  |  |  |
| **acute appendicitis** | |  |  |  |  |  |  |  |
| SNRI |  |  |  |  |  |  |  |  |
| with | 6658 | 2 |  |  |  |  |  |  |
| †without | 698 863 | 147 |  |  |  |  |  |  |
| SSRI |  |  |  |  |  |  |  |  |
| with | 25 402 | 6 | 0.94 | (0.37-2.38) | 0.94 | (0.38-2.38) | 1.13 | (0.44-2.90) |
| †without | 680 119 | 143 | 0.84 | (0.71-0.99) | 0.84 | (0.69-1.02) | 1 |  |
| TCA |  |  |  |  |  |  |  |  |
| with | 25 135 | 5 | 0.79 | (0.33-1.92) | 0.80 | (0.33-1.92) | 0.91 | (0.37-2.23) |
| †without | 680 386 | 144 | 0.85 | (0.72-1.00) | 0.88 | (0.73-1.05) | 1 |  |
| TeCA |  |  |  |  |  |  |  |  |
| with | 19 225 | 3 |  |  |  |  |  |  |
| †without | 686 296 | 146 |  |  |  |  |  |  |
| Others |  |  |  |  |  |  |  |  |
| with | 3522 | 0 |  |  |  |  |  |  |
| †without | 701 999 | 149 |  |  |  |  |  |  |

*Adjusted by inverse probability of treatment weighting using the propensity score (gender, age, medical utilization, hypertension, myocardial infarction, congestive heart failure, percutaneous coronary intervention, coronary bypass surgery, peripheral vascular disease, cerebrovascular disease, ischemic stroke, transient ischemic attack, hemiplegia or paraplegia, dementia, epilepsy, diabetes mellitus, chronic kidney disease, chronic pulmonary disease, peptic ulcer disease, liver disease, malignancy, anemia, rheumatic disease, human immunodeficiency virus infection, antibiotics and antifungal drugs, antiepileptics, antihypertensives, antiplatelets, bisphosphate, cardiovascular drugs, cyclosporine, glucocorticoid, insulin, lipid lower drugs, nonsteroid anti-inflammatory drugs, residence, income level, and occupation; see **Table 1** and **Supplementary Table 2-4**).

†without indicates DOAC alone.

**Supplementary Table 18.** Major bleeding risk among AF patients taking direct oral anticoagulants (DOACs) with or without concurrent anti-depressants, after removing bleeding events within the first person-quarter.

| **Concurrent medication** | **Person-Quarters with DOAC use** | **No. of Bleeding Events** | **Crude Major Bleeding Incidence Rate (95% CI) per 1000 Person-Years** | | ***Adjusted Incidence Rate (95% CI) per 1000 Person-Years** | | ***Adjusted Rate Ratio (95% CI)** | |
| --- | --- | --- | --- | --- | --- | --- | --- | --- |
| **Major bleeding** |  |  |  |  |  |  |  |  |
| SNRI |  |  |  |  |  |  |  |  |
| with | 6642 | 96 | 58.20 | (47.22-71.72) | 58.62 | (47.66-72.10) | 1.14 | (0.93-1.41) |
| †without | 698 879 | 7898 | 45.68 | (44.58-46.81) | 51.22 | (48.81-53.75) | 1 |  |
| SSRI |  |  |  |  |  |  |  |  |
| with | 25 344 | 328 | 51.68 | (46.13-57.91) | 52.30 | (46.74-58.53) | 1.02 | (0.91-1.15) |
| †without | 680 177 | 7666 | 45.58 | (44.47-46.72) | 51.19 | (49.68-52.76) | 1 |  |
| TCA |  |  |  |  |  |  |  |  |
| with | 25 083 | 318 | 50.82 | (45.29-57.03) | 51.23 | (45.69-57.46) | 1.02 | (0.91-1.15) |
| †without | 680 438 | 7676 | 45.61 | (44.50-46.75) | 50.17 | (48.71-51.69) | 1 |  |
| TeCA |  |  |  |  |  |  |  |  |
| with | 19 179 | 265 | 55.24 | (48.63-62.74) | 55.98 | (49.34-63.51) | 1.07 | (0.94-1.22) |
| †without | 686 342 | 7729 | 45.54 | (44.43-46.67) | 52.29 | (50.81-53.81) | 1 |  |
| Others |  |  |  |  |  |  |  |  |
| with | 3511 | 40 | 45.88 | (33.37-63.07) | 46.30 | (33.81-63.41) | 0.95 | (0.69-1.31) |
| †without | 702 010 | 7954 | 45.80 | (44.70-46.93) | 48.62 | (47.17-50.12) | 1 |  |
| **Intracerebral hemorrhage** | | |  |  |  |  |  |  |
| SNRI |  |  |  |  |  |  |  |  |
| with | 6642 | 13 | 8.05 | (4.69-13.80) | 7.94 | (4.61-13.66) | 1.05 | (0.61-1.83) |
| †without | 698 879 | 1208 | 6.99 | (6.59-7.41) | 7.52 | (6.96-8.13) | 1 |  |
| SSRI |  |  |  |  |  |  |  |  |
| with | 25 44 | 74 | 11.70 | (9.24-14.81) | 11.84 | (9.38-14.96) | #1.4 | (1.09-1.79) |
| †without | 680 177 | 1147 | 6.82 | (6.43-7.25) | 8.47 | (7.85-9.15) | 1 |  |
| TCA |  |  |  |  |  |  |  |  |
| with | 25 083 | 53 | 8.52 | (6.51-11.15) | 8.50 | (6.50-11.12) | 1.19 | (0.91-1.57) |
| †without | 680 438 | 1168 | 6.95 | (6.54-7.37) | 7.12 | (6.67-7.60) | 1 |  |
| TeCA |  |  |  |  |  |  |  |  |
| with | 19 179 | 50 | 10.44 | (7.86-13.86) | 10.51 | (7.93-13.93) | #1.33 | (0.99-1.77) |
| †without | 686 342 | 1171 | 6.90 | (6.51-7.33) | 7.92 | (7.39-8.49) | 1 |  |
| Others |  |  |  |  |  |  |  |  |
| with | 3511 | 4 | 4.57 | (1.68-12.44) | 4.64 | (1.74-12.39) | 0.59 | (0.22-1.57) |
| †without | 702 010 | 1217 | 7.01 | (6.61-7.44) | 7.93 | (7.31-8.59) | 1 |  |
| **Gastrointestinal hemorrhage** | | |  |  |  |  |  |  |
| SNRI |  |  |  |  |  |  |  |  |
| with | 6642 | 79 | 47.69 | (37.77-60.22) | 48.19 | (38.27-60.68) | 1.15 | (0.90-1.45) |
| †without | 698 879 | 6411 | 37.03 | (36.04-38.06) | 42.05 | (39.73-44.51) | 1 |  |
| SSRI |  |  |  |  |  |  |  |  |
| with | 25 344 | 248 | 39.01 | (34.20-44.49) | 39.47 | (34.66-44.95) | 0.96 | (0.84-1.10) |
| †without | 680 177 | 6242 | 37.06 | (36.06-38.10) | 41.06 | (39.70-42.46) | 1 |  |
| TCA |  |  |  |  |  |  |  |  |
| with | 25 083 | 260 | 41.42 | (36.39-47.15) | 41.85 | (36.80-47.59) | 1.01 | (0.89-1.15) |
| †without | 680 438 | 6230 | 36.98 | (35.97-38.01) | 41.40 | (40.02-42.82) | 1 |  |
| TeCA |  |  |  |  |  |  |  |  |
| with | 19 179 | 206 | 42.93 | (37.22-49.52) | 43.47 | (37.74-50.06) | 1.02 | (0.88-1.17) |
| †without | 686 342 | 6284 | 36.97 | (35.97-38.00) | 42.74 | (41.38-44.14) | 1 |  |
| Others |  |  |  |  |  |  |  |  |
| with | 3511 | 36 | 41.36 | (29.56-57.85) | 41.60 | (29.84-57.99) | 1.07 | (0.76-1.49) |
| †without | 702 010 | 6454 | 37.11 | (36.12-38.14) | 39.02 | (37.73-40.35) | 1 |  |
| **Intraspinal, intraocular, retroperitoneal, intra-articular, pericardial, or intramuscular hemorrhage** | | | | | | |  |  |
| SNRI |  |  |  |  |  |  |  |  |
| with | 6642 | 4 | 2.41 | (0.90 6.42) | 2.41 | (0.90-6.42) | 1.51 | (0.56-4.06) |
| †without | 698 879 | 279 | 1.6 | (1.42-1.80) | 1.60 | (1.37-1.88) | 1 |  |
| SSRI |  |  |  |  |  |  |  |  |
| with | 25 344 | 6 | 0.95 | (0.43-2.11) | 0.95 | (0.43-2.11) | 0.58 | (0.26-1.30) |
| †without | 680 177 | 277 | 1.63 | (1.44-1.84) | 1.64 | (1.42-1.89) | 1 |  |
| TCA |  |  |  |  |  |  |  |  |
| with | 25 083 | 5 | 0.80 | (0.33-1.91) | 0.80 | (0.33-1.91) | 0.51 | (0.21-1.23) |
| †without | 680 438 | 278 | 1.63 | (1.45-1.84) | 1.57 | (1.38-1.79) | 1 |  |
| TeCA |  |  |  |  |  |  |  |  |
| with | 19 179 | 9 | 1.88 | (0.98-3.60) | 1.88 | (0.98-3.60) | 1.21 | (0.62-2.35) |
| †without | 686 342 | 274 | 1.60 | (1.41-1.80) | 1.56 | (1.36-1.78) | 1 |  |
| Others |  |  |  |  |  |  |  |  |
| with | 3511 | 0 |  |  |  |  |  |  |
| †without | 702 010 | 283 |  |  |  |  |  |  |

*Adjusted by inverse probability of treatment weighting using the propensity score (gender, age, medical utilization, hypertension, myocardial infarction, congestive heart failure, percutaneous coronary intervention, coronary bypass surgery, peripheral vascular disease, cerebrovascular disease, ischemic stroke, transient ischemic attack, hemiplegia or paraplegia, dementia, epilepsy, diabetes mellitus, chronic kidney disease, chronic pulmonary disease, peptic ulcer disease, liver disease, malignancy, anemia, rheumatic disease, human immunodeficiency virus infection, antibiotics and antifungal drugs, antiepileptics, antihypertensives, antiplatelets, bisphosphate, cardiovascular drugs, cyclosporine, glucocorticoid, insulin, lipid lower drugs, nonsteroid anti–inflammatory drugs, residence, income level, and occupation, see **Table1** and **Supplementary Table 2-4**).

†without indicates DOAC alone.

#*P* < 0.05, compared with DOAC alone.

**Supplementary Fig. 1.** Schematic presentation of person-quarter design

***
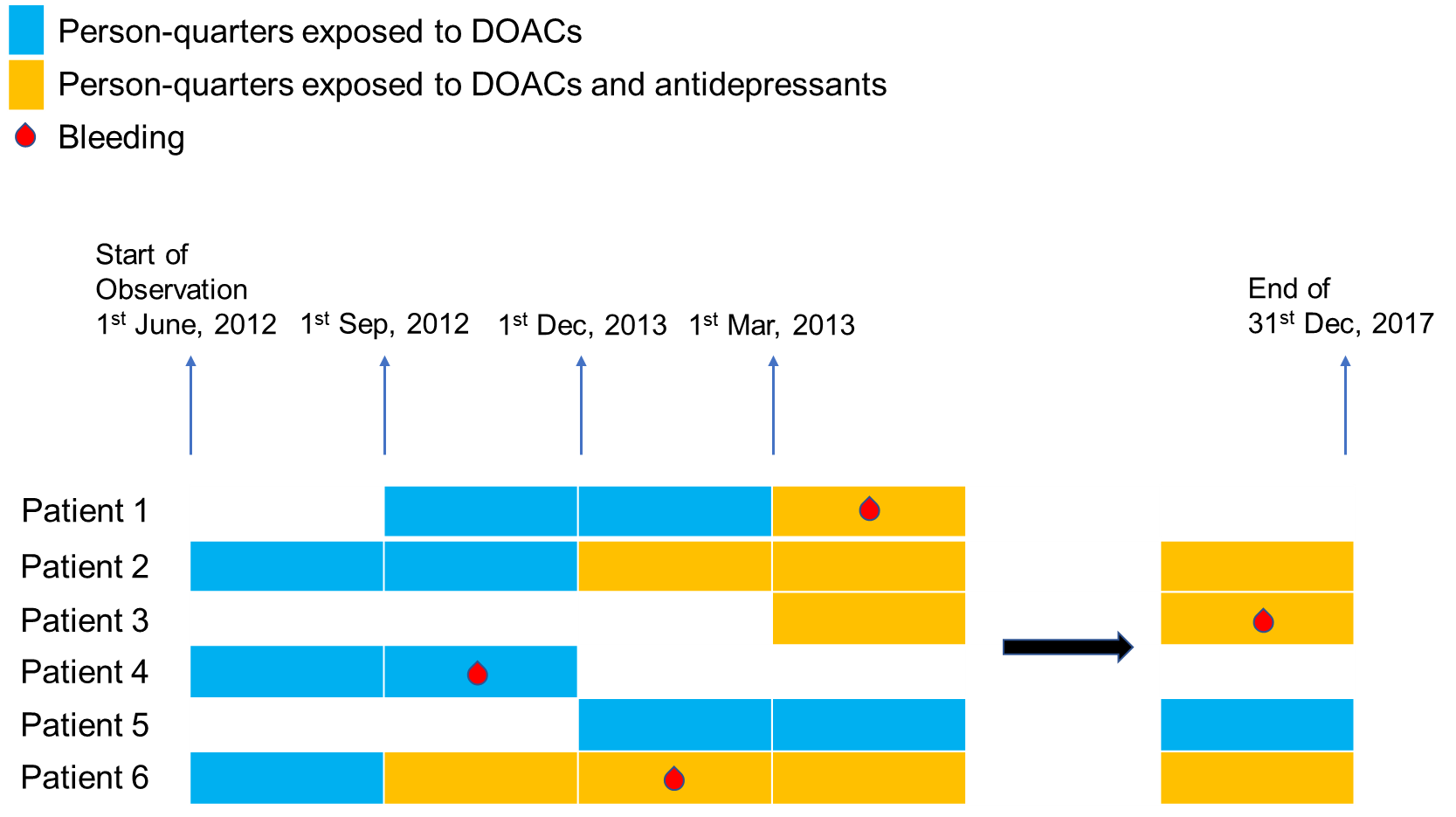
***
